# Cognitive Remediation Interventions for Gambling Disorder: A Systematic Review

**DOI:** 10.3389/fpsyg.2017.01961

**Published:** 2017-12-04

**Authors:** Gaëlle Challet-Bouju, Mélanie Bruneau, IGNACE Group, Marie Grall-Bronnec, Caroline Victorri-Vigneau, Marie Grall-Bronnec

**Affiliations:** (CHU Nantes/INSERM UMR 1246, France), (CHU Nantes/INSERM UMR 1246, France), (CHU Nantes/INSERM UMR 1246, France), (CHU Nantes/INSERM UMR 1246, France), (CHU Nantes/INSERM UMR 1246, France), (University of British Columbia, Canada), (CNRS UMR 5229, France), (The Netherlands), (University of Amsterdam/Arkin Mental Health Care, The Netherlands), (CNRS USR 3413, France), (University of Luxembourg, Luxembourg), (Hôpitaux Universitaires de Genève/University of Geneva EA834-PSY, Switzerland), (Bellvitge University Hospital, Spain), (Sainte Anne Hospital/University Paris Descartes/INSERM U894, France), (Sainte Anne Hospital/University Paris Nanterre/INSERM U894, France), (Université Laval, Québec), and (University of Sydney, Australia).; ^1^Clinical Investigation Unit “Behavioral Addictions/Complex Affective Disorders”, Department of Addictology and Psychiatry, CHU Nantes, Nantes, France; ^2^Université de Nantes, Université de Tours, Institut National de la Santé et de la Recherche Médicale (INSERM) UMR 1246 SHERE, Nantes, France; ^3^Department of Pharmacology, Center for Evaluation and Information on Pharmacodependence, CHU Nantes, Nantes, France

**Keywords:** gambling, cognitive remediation, cognitive dysfunction, cognitive bias modification, therapy

## Abstract

Various therapeutic approaches are available for the treatment of gambling disorder (GD), especially cognitive behavioral therapy (CBT; the most widely used treatment). However, CBT has high dropout and relapse rates as well as non-compliance issues, which may be partly due to resistance to changing core characteristics, such as executive functioning, attention, and emotional regulation abnormalities. Finding new therapeutic approaches to treat GD is thus a key challenge. Cognitive remediation (CR) interventions represent a promising approach to GD management, which has recently been demonstrated to have efficacy for treating other addictive disorders. The objective of this review is to describe the possible benefits of CR interventions for GD management. Two systematic searches in MEDLINE and ScienceDirect databases were conducted up until January 2017. Potential neurocognitive targets of CR interventions for GD were reviewed, as is the use and efficacy of such interventions for GD. While there is evidence of several neurocognitive deficits in individuals with GD in terms of impulsive, reflective, and interoceptive processes, the literature on CR interventions is virtually absent. No clinical studies were found in the literature, apart from a trial of a very specific program using Playmancer, a serious videogame, which was tested in cases of bulimia nervosa and GD. However, neurocognitive impairments in individuals with addictive disorders are highly significant, not only affecting quality of life, but also making abstinence and recovery more difficult. Given that CR interventions represent a relatively novel therapeutic approach to addiction and that there is currently a scarcity of studies on clinical populations suffering from GD, further research is needed to examine the potential targets of such interventions and the effectiveness of different training approaches. So far, no consensus has been reached on the optimal parameters of CR interventions (duration, intensity, frequency, group vs. individual, pencil-and-paper vs. computerized delivery, etc.). Although no firm conclusions can be drawn, CR interventions represent a promising adjunct treatment for GD. Such a novel therapy could be associated with common interventions, such as CBT and educational and motivational interventions, in order to make therapies more effective and longer-lasting and to decrease the risk of relapse.

## Introduction

Gambling Disorder (GD) is defined as a “persistent and recurrent problematic gambling behavior leading to clinically significant impairment or distress” (American Psychiatric Association, 2013). Included in the spectrum of addictive disorders in the 5th version of the Diagnostic and Statistical Manual of Mental Disorders (DSM-5), GD shares many similarities with substance use disorders (SUD), at the behavioral, psychological, and neurobiological level (Reilly and Smith, 2013). The prevalence of lifetime GD has been estimated at around 0.4–1.0% (American Psychiatric Association, 2013).

Various therapeutic approaches are available for the treatment of GD, which include psychological interventions (cognitive behavioral therapy, motivational interviewing; Merkouris et al., 2016), mindfulness (de Lisle et al., 2012), pharmacological medications (opioid antagonists (Victorri-Vigneau et al., 2017), glutamate agonists, antidepressant drugs, mood stabilizers; Grant et al., 2014), self-help and peer-support (Merkouris et al., 2016). Recently, novel and promising treatment options have also been explored, such as Virtual Reality (Giroux et al., 2013) and neuromodulation (repeated Transcranial Magnetic Stimulation; Grall-Bronnec and Sauvaget, 2014 or Transcranial Direct Current Stimulation; Sauvaget et al., 2015). Psychological interventions, especially cognitive behavioral therapy (CBT), appear to be the most widely used treatment for the management of GD with demonstrated efficacy (Korn and Shaffer, 2004; Gooding and Tarrier, 2009; Stea and Hodgins, 2011). However, the extent and durability of effectiveness remains unclear (Cowlishaw et al., 2012) and CBT are associated with high dropout rates, relapses, and non-compliance issues (Jimenez-Murcia et al., 2012; Goudriaan et al., 2014; Tarrega et al., 2015; Merkouris et al., 2016). This might be partly due to resistance to change of several core characteristics in GD, such as executive functioning, attention, and emotional regulation (self-control strategies, tolerance to frustration, and impulsivity traits) (Merkouris et al., 2016). Finding novel therapeutic approaches for the treatment of GD is a key challenge, especially those that can target patients with more severe symptoms, high levels of impulsivity and impaired emotional regulation.

Cognitive remediation (CR) interventions represent a specific neuropsychological treatment aimed at improving cognitive functioning, in order to reduce the impact of a disease in a patient's life. They have been defined as “*a behavioral training based intervention that aims to improve cognitive processes (attention, memory, executive function, social cognition, or metacognition) with the goal of durability and generalization*” (Barlati et al., 2013; Medalia and Bowie, 2016). Therefore, in contrast to CBT, the primary goal of CR interventions is to improve the thinking process rather than the content of thoughts. CR interventions are based on the neuroplasticity hypothesis, which states that the brain has an inherent capacity to change and reorganize dependent on our experiences throughout life. CR interventions are expected to induce neuroplastic changes through the use of targeted cognitive exercises and training, either using “paper and pencil” or computerized exercises, leading to concomitant cognitive/behavioral changes that could be transferred into clinically relevant benefits for the patients (in terms of disease symptoms, autonomy, or social interactions) (Mishra and Gazzaley, 2014).

CR interventions consist of various techniques and methods, with the common aim of restoring neurocognitive abilities and/or compensating for impairments in them. To date, most clinical experiences and research findings have focused on schizophrenia and, overall, three major types of CR interventions have emerged over the past 20 years (that are not mutually exclusive) (Medalia and Bowie, 2016):

1. The compensatory/strategy-based approach, which focuses on counteracting cognitive difficulties by acquiring new and efficient skills to transfer to the real world, and modifying the local environment to foster the successful completion of activities in everyday life. Using cognitive exercises, programs may target different skills, such as cognitive flexibility, memory, and planning. This approach attempts to recruit intact cognitive processes in order to bypass cognitive deficits and improve targeted behaviors and functional outcomes.
2. The restorative approach has an underlying assumption that improvements in cognition are mediated by neuroplasticity. This approach targets cognitive impairments directly through repeated task practice, careful titration of task difficulty, and maintenance of high levels of accurate performance. It is usually computer assisted.
3. The social cognitive approach, which focuses on ameliorating deficits in taking others' perspectives (theory of mind) into consideration, improving alterations in recognizing expressed affect, and retraining information processing biases. These programs are specifically designed for patients with schizophrenia who present with multiple impairments in social cognition.

CR interventions have been applied to many neurocognitive disorders, including Alzheimer's disease (Bahar-Fuchs et al., 2013), schizophrenia (Paquin et al., 2014), multiple sclerosis (O'Brien et al., 2008), Parkinson disease (Nombela et al., 2011), and depression (Calkins et al., 2015). There is strong evidence to support their efficacy (Rezapour et al., 2016). The interest in using such interventions in the treatment of addiction has recently emerged, due to their expected therapeutic effects and potential to regain control over addictive behavior, especially by enhancing inhibitory control (Sofuoglu et al., 2013). CR interventions represent a promising option for the care of addicts, and have already demonstrated efficacy in the treatment of alcohol dependence (Rupp et al., 2012) and drug addictions (Sofuoglu et al., 2013). They could be integrated with other addiction treatments using a holistic and patient-centered approach (Rezapour et al., 2016), and must be adapted by targeting either only one or multiple cognitive functions (Rezapour et al., 2015) to correspond with the specific neurocognitive needs of individual patients (Bayley et al., 2014).

The cognitive alterations of individuals with GD have been the subject of multiple studies and reviews (Goudriaan et al., 2004; Brevers and Noël, 2013; Hønsi et al., 2013). In particular, these studies have been conducted from 2000 to the present, supporting the grouping of GD within the framework of addictive disorders, as they were previously restricted to SUD before the publication of the DSM-5 (American Psychiatric Association, 2013). They identified several common neuropsychological deficits between those with GD and SUD, especially for executive functioning and attentional biases [comparison with cocaine-dependent individuals (Albein-Urios et al., 2012); comparison with alcohol-dependent individuals (Goudriaan et al., 2006a), comparison with methamphetamine-dependent individuals (Kalechstein et al., 2007)].

The objective of this review is to describe the potential benefits of CR interventions for the management of GD. It includes an updated review of cognitive alterations as potential neurocognitive targets in CR interventions for GD, and a review of the use and efficacy of such interventions for the treatment of individuals with GD.

## Methods

Two systematic reviews of the literature were conducted to identify all the relevant publications concerning:

1. Potential neurocognitive targets of CR interventions for GD management. The aim was to identify the major neurocognitive processes altered in individuals with GD that could be targeted by CR interventions. We should emphasize that this first review was made to provide support for the use of CR interventions in GD management, and was not aimed at identifying the neurocognitive mechanisms underlying the development or maintenance of GD.
2. The use and efficacy of CR interventions for the treatment of GD. The aim was to explore whether literature exists on the use and efficacy of such interventions for individuals with GD.

For both of these reviews, we complied with the Preferred Reporting Items for Systematic reviews and Meta-Analyses (PRISMA) (Moher et al., 2009).

### Search strategy

The searches were performed in MEDLINE and ScienceDirect databases up until January 17th 2017 and were limited to articles published in English. For the first review on potential targets for CR interventions in GD, the search terms were a combination of medical subject headings (MeSH) terms and keywords including: “pathological gambling,” “problem gambling,” “gambling disorder,” “gambling addiction” AND “cognitive functions,” “cognitive dysfunction,” “executive function,” “memory disorders,” “neurocognitive disorders,” “attention,” “cognitive impairment.”

For the second review on the use and efficiency of CR interventions in GD, the search terms were also a combination of MeSH terms and keywords including: “gambling,” “pathological gambling,” “problem gambling,” “gambling disorder,” “gambling addiction” AND “cognitive remediation therapy,” “cognitive training,” “cognitive rehabilitation therapy,” “cognitive retraining,” “cognitive bias modification,” “executive training,” “cognitive remediation,” “cognitive reappraisal.”

A manual search and screening of the bibliographic references of the studies included were performed in addition to the database search.

**Figure 1:**
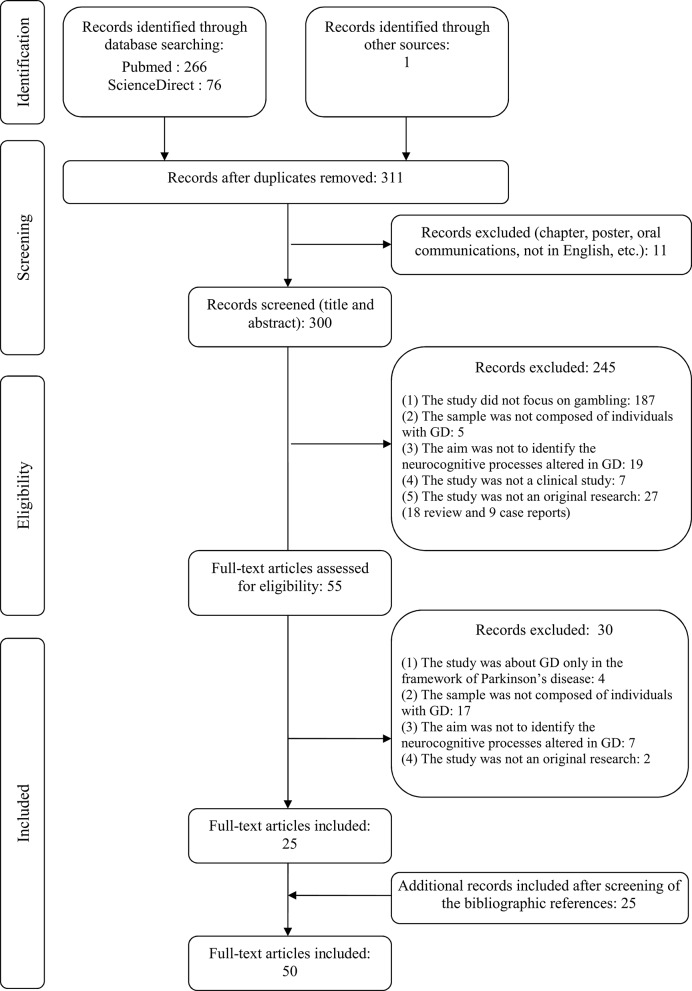
Flow diagram of the first review on potential neurocognitive targets of Cognitive Remediation (CR) interventions for Gambling Disorder (GD) management.

**Figure 2:**
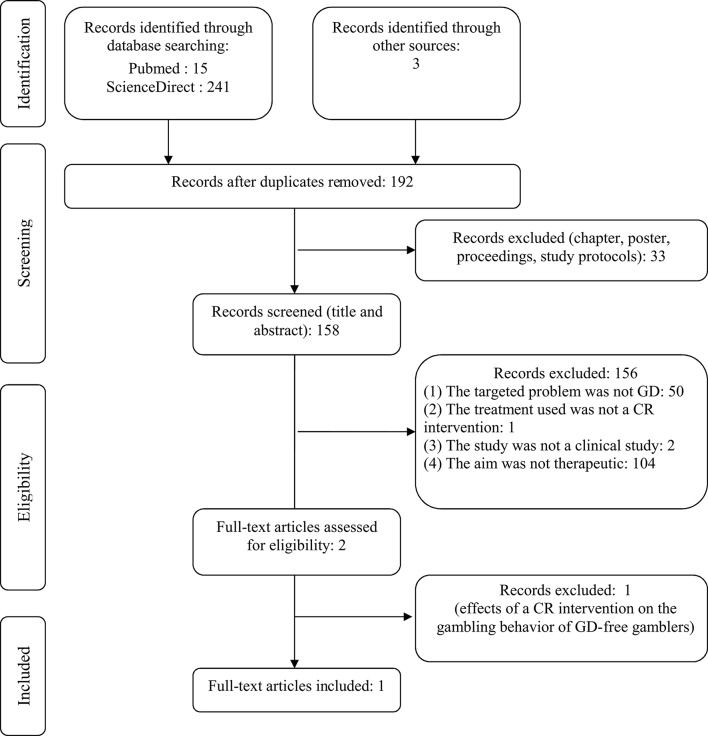
Flow diagram of the second review on the use and efficacy of Cognitive Remediation (CR) interventions for the treatment of Gambling Disorder (GD).

Flow diagrams of the two systematic review processes are presented in Figures 1, 2.

### Eligibility criteria

For the first review on potential targets for CR interventions in GD, studies had to fulfill the following criteria to be included:

1. The study focused on gambling.
2. The sample was composed of individuals with GD. As the definition of pathological gambling has largely evolved during recent years, we included studies on both problem/excessive gambling [as defined by a score of 5 or more at the South Oaks Gambling Screen (SOGS) (Lesieur and Blume, 1987) or a score of 8 or more at the Problem Gambling Severity Index (PGSI) (Ferris and Wynne, 2008)] and diagnosed GD (according to the DSM or the International Classification of Diseases [ICD] diagnostic criteria). We thus excluded studies conducted on subthreshold forms of problem gambling (especially studies using a threshold of under 5 on the SOGS or under 8 on the PGSI, or with less than the required number of DSM diagnostic criteria) and studies conducted on self-identified problem gamblers. We made this choice because CR interventions are directed toward patients with a confirmed GD or problem/excessive gambling practice.
3. The aim of the study was to identify the neurocognitive impairments related to GD (for example, attention deficits, altered executive functioning, or memory disorders), which are part of the endophenotype and may be the target of specific CR interventions. As a consequence, only studies that have at least one neurocognitive measure as an outcome were included. We excluded from this review the clinical expression of certain cognitive dimensions often measured with questionnaires, which are part of the exophenotype and the targets of CBT, such as gambling-related cognitive distortions and impulsivity (understood as a personality trait).
4. The study was a clinical study (e.g., any research study involving human volunteers intended to add to medical knowledge, including pilot studies, observational studies, and randomized clinical trials) (U.S. and National Institutes of Health, 2017).
5. The study was original research (not a case report, editorial article, or a review).

For the second review on the use and efficiency of CR interventions in GD, studies had to fulfill the following criteria to be included:

1. The target problem was a GD.
2. The sample was composed of individuals with GD.
3. The study had a therapeutic aim (for example, efficiency or effects of the CR interventions on individuals with GD).
4. The study investigated an intervention that can be classified as a CR intervention. This comprises any type of compensatory, restorative, or social-cognitive approaches.
5. The study was a clinical study (U.S. and National Institutes of Health, 2017).

### Study selection and data extraction

All studies were screened for eligibility based on their titles and abstracts by the first and last authors (GCB and MGB) for the first review and by the first two authors (GCB and MB) for the second review. Reasons of exclusion are reported in the flow diagrams (Figures 1, 2). Then, the full texts of all eligible studies identified in the search processes were read and several pieces of information were extracted: sample size and participants, mean age of participants, main exclusion criteria, objectives, design, tasks, or questionnaires used to measure neurocognitive functions, main results, and limitations.

## Results

### First review on potential targets for CR interventions in GD

As depicted in Figure 1, 50 studies fulfilled all the criteria to be included in the review. Studies are reported in chronological order, in order to highlight changes in the methods used or results obtained over time.

We observed that the high number of studies wrongly identified by the database search was due to the huge use of the Iowa Gambling Task (IGT) in the scientific literature to assess decision-making related processes in a large number of pathologies. As the IGT includes the word “Gambling” in its name, the database search initially led to an overinclusion of studies.

The methods of all studies included are presented in Supplementary Table 1. The oldest study was conducted in 1995, but the large majority of the studies were conducted between 2000 and the present. This exploration of neurocognitive deficits in individuals with GD is quite recent, and was especially accentuated with the preparation of the DSM-5. Of the 50 studies included, only one had a longitudinal design (Goudriaan et al., 2008), with the aim of finding neurocognitive predictors of relapse. The absence of other longitudinal studies raises the question of the maintenance of neurocognitive alterations in individuals with GD over time, and of their effects on treatment outcomes and relapses. The studies included were predominantly conducted on males and used low sample sizes, with nearly half of them (45%) being conducted on less than 30 individuals with GD. This could be due to the difficulty of conducting in-depth neurocognitive assessments on a large sample. However, as neurocognitive studies often include a large number of dependent variables, a low sample size may result in biased conclusions. Also, inclusion criteria varied a lot and specifically, assessment tools and thresholds used to include individuals with GD varied highly, from a SOGS score over or equal to 5 to a real clinical diagnosis of GD. Studies were conducted on a large range of GD severity, and mixed problem gamblers (PrG) and pathological gamblers (PG) (for details see Supplementary Table 1). This may have led to results being biased and/or limited. Another factor bringing possible bias to the results is the fact that most of the studies compared PG or PrG to non-gambler controls (see Supplementary Table 1 for more details). In this condition, it is hard to identify whether the alterations identified are related to gambling itself or to GD-related psychopathology.

The main results of this review are synthetized in Supplementary Table 2. We have only presented studies that compared PrG or PG, to healthy controls or non-problem gamblers, in order to identify only those neurocognitive alterations that are due to specific gambling psychopathology (forty-four studies of the fifty initially included). To facilitate understanding, the various cognitive functions assessed in the studies included were grouped within eight categories: (1) general cognitive functioning; (2) memory and working memory; (3) attention; (4) cue-reactivity for gambling cues; (5) metacognition; (6) executive functioning with six sub-categories (response inhibition, concept generation and abstraction, planning, time estimation, flexibility, and decision-making); (7) social cognition; and (8) visuo-spatial and visuo-constructive abilities. This presentation is obviously simplistic and the grouping of cognitive functions is debatable, as one function cannot be assessed purely by one cognitive task and because certain high-level functions require the involvement of others. It has only one objective—to be clearer.

General cognitive functioning appeared to be preserved in most cases, as was the capacity for memory. Visuo-spatial and visuo-constructive abilities appeared alerted compared to controls, although these alterations were assessed in only two studies (Forbush et al., 2008; Hur et al., 2012).

Specific assessment of attention capacities is relatively rare in the literature and only two studies where this was addressed were identified in the present review (Vizcaino et al., 2013; Lorains et al., 2014b). This is considerably less than in the review that focused on attentional biases in problem gambling conducted in 2013 by Hønsi et al. (2013), who identified 11 studies, but with no restriction on the threshold to identify problem or pathological gamblers. In the two studies selected for the present review, results are divergent. While Lorains et al. (2014b) found no differences between individuals with GD and controls, the introduction of gambling-related cues as stimuli for assessing the maintenance of attention-induced attentional bias, showed no correlation between PG severity and degree of attentional bias (Vizcaino et al., 2013). In the framework of the present review, only one study by Sharpe et al. involved examining cue-reactivity for gambling cues in individuals with GD (Sharpe et al., 1995). Other studies on cue reactivity in GD were performed with the aim of identifying brain regions involved in craving (Limbrick-Oldfield et al., 2017), which was considered outside the scope of the present review. In their study, Sharpe et al. concluded there is a higher influence of gambling-related cues on physiological arousal (measured by skin conductance levels, frontalis electromyography, and electrocardiography) in individuals with GD compared to non-problem gamblers, even high-frequency non-problem gamblers, although it was conducted on a small sample size. These effects were limited when a cognitive distraction task was added, especially for individuals with GD, suggesting that competing thoughts are useful when confronted with gambling stimuli.

Metacognitive judgement was examined in only two studies, and this was mainly by assessment of the level of confidence in various risky choices, both in (Brevers et al., 2013) and out (Goodie, 2005) of a gambling context. Outside of a gambling context, individuals with GD displayed greater overall overconfidence and bet acceptance (Goodie, 2005). In an experimental gambling situation (the IGT), individuals with GD were more confident than controls for disadvantageous decisions, but not for advantageous ones (Brevers et al., 2013), leading to a recurrent higher tendency to make disadvantageous choices and, consecutively, to lower performances in this task (Cavedini et al., 2002; Goudriaan et al., 2005, 2006b; Lakey et al., 2007; Forbush et al., 2008; Roca et al., 2008; Kertzman et al., 2011; Ledgerwood et al., 2012; Brevers et al., 2014; Lorains et al., 2014a).

What can clearly be concluded from Supplementary Table 2 is that the majority of the research to date has been focused on executive functioning (93% of the 44 distinct studies presented in Supplementary Table 2), especially on decision-making (57%), and to a lesser extent on response inhibition (50%). Response inhibition has been assessed within two modalities: cognitive inhibition (interference control) and motor inhibition (inhibition of a prepotent response). Cognitive inhibition has been assessed classically with the Stroop test, and predominantly with the classical word and color Stroop test (11 studies out of 12). Only one study used an addiction version of the Stroop test, which has the same principles as the classical Stroop, but with addiction-related stimuli. Results were divergent. Of the 12 studies identified, only seven identified alterations in cognitive inhibition based on the Stroop task, but three of the five studies that did not pick up any difference involved very small sample sizes, or aimed to compare GD to other pathologies [one study was of a comparison with bulimia nervosa and included 15 PG (only females) (Alvarez-Moya et al., 2009); one study was of a comparison with obsessive compulsive disorder and included 16 PG (Hur et al., 2012); one study included 13 PG (only males) (Potenza et al., 2003)]. More specifically and unexpectedly, the only study that used an addiction version of the Stroop test (Lorains et al., 2014b) did not find any differences between inhibition performance in PrG and controls. However, the task was programmed in such a way that the cognitive response was recorded through a motor response rather than a vocal response, which may have induced bias. Motor inhibition was assessed with both Go/No Go or related paradigms (GNG paradigms—six studies identified) and Stop Signal Task or related paradigms (SS paradigms—eight studies identified). These tasks measure different components of motor inhibition: the GNG paradigms assess inhibition of the initiation of a motor response with automatic inhibition likely to occur, whereas the SS paradigms assess the interruption of an on-going motor response with automatic inhibition unlikely to occur (Verbruggen and Logan, 2008; Billieux et al., 2014). Out of the six studies conducted with GNG paradigms, five reported alterations in motor inhibition (Goudriaan et al., 2005; Fuentes et al., 2006; Kertzman et al., 2008, 2011; Roca et al., 2008). The only study that did not identify any difference between individuals with GD and controls was performed using a reward-punishment version of the GNG paradigm, with incentives for learning given for every correct response, and on a small sample size (Leiserson and Pihl, 2007). The alterations identified mainly concerned the number of errors (both omission and commission errors) and response times to Go trials, with both faster (Goudriaan et al., 2005; Roca et al., 2008) or longer (Kertzman et al., 2008, 2011) reaction times recorded. This varying effect on reaction times may be explained by the higher variability in reaction times in individuals with GD than in controls (Kertzman et al., 2008). Only half of the eight studies based on SS paradigms identified an alteration in motor inhibition, although the four studies with negative results (Ledgerwood et al., 2009, 2012; de Ruiter et al., 2012; Lorains et al., 2014b) were conducted on PG rather than PrG, possibly indicating a GD of higher severity. Indeed, Odlaug et al. demonstrated that performance at the SST was poorer for PG than for at-risk gamblers or non-problem gamblers, whereas at-risk gamblers displayed the same level of performance as non-problem gamblers (Odlaug et al., 2011). When an effect was observed, lower motor inhibition was associated with longer Stop Signal Reaction Time (SSRT) (Goudriaan et al., 2006a; Odlaug et al., 2011; Billieux et al., 2012; Grant et al., 2012b) and, to a lesser extent, longer Go reaction times (Odlaug et al., 2011). Importantly, in the one longitudinal study, the lowering of SSRT, which is indicative of a poor capacity for motor inhibition, was associated with a higher likelihood of relapse 1 year after treatment (Goudriaan et al., 2008). It seems that alterations in motor inhibition are related more to the difficulty in inhibiting initiation of an action, rather than to the difficulty of stopping an action once initiated. Making a parallel with gambling behavior, it is presumably harder for gamblers to avoid engaging in a gambling action than to interrupt it once initiated.

The large part of the neuropsychological studies on GD has been concerned with decision-making abilities. As illustrated in Supplementary Table 2, there is no doubt a decision-making deficit exists in individuals with GD. This deficit can take the form of: (1) delay discounting difficulties with a lower ability to delay rewards; (2) lower impact of negative feedback on future decisions; (3) sensitivity to monetary reward and punishment with higher cognitive and physiological sensitivities to gains and, to a lesser extent, lower cognitive, and physiological sensitivities to losses; (4) impaired risk assessment with altered anticipatory physiological reactions to risky decision-making, and (5) a general trend toward making disadvantageous risky and/or ambiguous choices, even when no monetary rewards are involved (Linnet et al., 2006). From Supplementary Table 2 it can be seen that the task predominantly used to assess decision-making capacities was IGT (44% of the 25 distinct studies on decision-making, compared to 4–12% for the other tasks), which assesses both decision-making under ambiguity (throughout the beginning of the task, the patient does not have conscious knowledge of which are the good decks, and makes choices under uncertainty) and risk (after several trials, the patient gradually acquires conscious knowledge of which are the good decks and thus can consciously make risky choices). Individuals with GD display lower global performance, and no shift toward advantageous card selection during the task compared to controls. It appears that alterations in decision-making abilities are only present in a gambling context (typically, the IGT) or, at least, when monetary rewards are involved (Billieux et al., 2012), but not outside of these contexts (Ledgerwood et al., 2009; Fauth-Bühler et al., 2014). Motivational aspects (especially of a monetary kind) of decision-making are thus of crucial importance, and may be more automatized and difficult to control in individuals with GD than “cold” reflective ones, which appear to be preserved. These decision-making deficits are all the more important in that they are predictors of the probability of relapse 1 year after treatment (Goudriaan et al., 2008).

Concept generation and abstraction has been largely assessed in GD (16% of the 44 distinct studies presented in Supplementary Table 2), especially using the Wisconsin Card Sorting Test (WCST) (86% of the studies exploring concept generation and abstraction). This test, which can serve as a measure of general executive functioning and of reactive flexibility, can also constitute an index of concept generation and abstract reasoning by mainly utilizing the number of categories completed, number of non-perseverative errors, learning-to-learn score and percentage of conceptual responses. In most cases, individuals with GD display a similar number of completed categories as controls, but altered learning-to-learn scores and percentages of conceptual response scores. This might indicate a preserved global performance, but difficulty with concept generation. Results on non-perseverative errors are more mitigated.

Flexibility can be assessed in two ways: reactive flexibility (the ability to adapt strategies dependent on feedback from the environment) and spontaneous flexibility (the ability to spontaneously produce a flow of ideas, with no feedback from the environment). In the selected studies (*n* = 10), reactive flexibility was assessed mainly both with the WCST (a higher number of perseverative errors indicating a poorer flexibility) (60%) and the Trail Making Test (TMT) (30%). In both tests divergent results were obtained, with half of the studies producing negative results in the WCST and two out of three studies doing so in the TMT. Spontaneous flexibility was mainly assessed with fluency tests [the Controlled Oral Word Association Test being the most used (67%)]. As for reactive flexibility, results were divergent, with half of the studies producing negative results. As can be seen in Supplementary Table 2, the results about flexibility is quite unstable over time, as more recent studies did not find deficits in both reactive and spontaneous flexibility.

Finally, other functions were assessed, but in a smaller number of studies, making it difficult to derive conclusions. Planning had been assessed in two studies, both using the Tower of London test (Goudriaan et al., 2006a; Ledgerwood et al., 2012) and concluding that there were altered planning abilities in individuals with PG. Estimation of time was also assessed, but in only one study (Goudriaan et al., 2006a), in which lower performance in individuals with PG than controls was demonstrated.

Only one study explored social cognition in GD (Kornreich et al., 2016). Using three emotion recognition tasks (musical, vocal, and facial), Kornreich et al. demonstrated that individuals with GD presented non-verbal perception deficits, in the same way as alcohol-dependent patients do. This represents the first study that explored social cognition deficits in GD. Unfortunately, the study had several limitations and requires repetition.

Finally, two studies explored visuo-spatial and visuo-constructive abilities, and both came to the conclusion that impairments were present (Forbush et al., 2008; Hur et al., 2012).

### Second review of the use and efficiency of CR interventions in GD

As depicted in Figure 2, despite the fact that the initial database search resulted in 192 records, only one study fulfilled all the criteria for inclusion in the review. The main reasons for exclusion were that the targeted problem was not GD (the fifty studies excluded were mainly about schizophrenia, neurodevelopmental disorders, neurodegenerative disorders, or attention deficit hyperactivity disorder) or that the aim was not therapeutic (the one-hundred and four studies excluded were mainly on the benefits of videogames for cognitive functioning, cognitive enhancement in healthy subjects, aging well, pedagogy, or productivity management, or were theoretical papers for modeling cognitive or neurobiological functioning in gambling or other domains).

We suppose that there are two reasons for the huge number of studies incorrectly identified by the database search. Firstly, CR interventions are often game-like exercises so that the use of the word “gambling” (often linked to the word “gaming”) within the search strategy could have led to an over-identification of studies not related to GD. Secondly, the majority of the studies excluded were about CR interventions on healthy subjects (to improve or take advantages of cognitive training-like exercises in everyday life), but our focus was on studies into the use of CR interventions as a therapeutic approach for patients with GD.

Due to studies on the use of CR interventions for addictions being quite recent in the literature, and because the use of such interventions is less obvious for GD than for substance-related addictions due to the absence of the neurotoxic effects of a psychoactive substance, there is a scarcity of studies on the use CR interventions in GD in the literature. Our review was, hence, inconclusive, and we failed to find any program or even any exercises where CR interventions had been applied to individuals with GD, apart from one study of a serious videogame (Playmancer) used in GD, in addition to CBT (Tarrega et al., 2015).

Serious videogames are not strictly part of CR interventions, but they are close in some ways. Playmancer is a serious video-game with biofeedback, designed to treat impulse control disorders (Jimenez-Murcia et al., 2009; Fernandez-Aranda et al., 2012; Tarrega et al., 2015). It has already been used in patients suffering from bulimia nervosa (Fagundo et al., 2013; Giner-Bartolome et al., 2015). This application may be referred to as a CR intervention, as the purpose of this technique was to improve emotional regulation and self-control, reducing arousal, and enhancing decision-making, and planning (Tarrega et al., 2015). CR interventions are mostly provided through computer-assisted technologies, and serious videogames have become an interesting way forwards for cognitive training being also innovative tools that are highly motivating for the majority of users. Preliminary results were interesting with a positive effect on impulsivity, expressions of anger and other psychopathological symptoms, but no evidence of any benefits in terms of dropout rates and relapses was observed (Tarrega et al., 2015). However, this technique is still novel and very few studies have been reported on its relevance to, and efficacy in treating, addictive behavior. The two studies (the one on bulimia nervosa and the one on GD) were uncontrolled and used small unrepresentative samples.

Another attempt to set up a CR program for gambling was proposed by Stevens et al. but this time in a sample of healthy volunteers (Stevens et al., 2015). As a consequence, this study was not included in the present review, but it should still be mentioned here. Stevens et al. stated that the training of motor inhibition, especially by including stop signals in a gambling task, influences gambling by reducing approach behavior and altering the motivational value of gambling outcomes (Stevens et al., 2015). Further research is needed to generalize these results to individuals with GD, but the results support the potential of CR interventions in managing GD.

## Discussion

The two reviews reported here have identified a paradox. While there is evidence of several neurocognitive deficits in individuals with GD, any literature on CR interventions is almost absent. Research into CR interventions on GD is just beginning and we expect there to be many more studies in future. There were no clinical trials found in the literature, apart from a report of the use of a very specific program using Playmancer, a serious videogame, tested in bulimia nervosa and GD (Fagundo et al., 2013; Tarrega et al., 2015). Yet, neurocognitive impairments in addicted patients are of great significance, not only affecting quality of life, but also making abstinence and recovery more difficult. In GD, these neurocognitive impairments lead to an increased risk of becoming, or remaining, addicted to gambling, but are also strong predictors of gambling relapse (Goudriaan et al., 2008). Therefore, it is very important to act on these impairments within the framework of care.

### What are the potential targets for CR interventions?

Since the early 2000s, the dual-process model of addiction has been the one largely developed (Strack and Deutsch, 2004; Evans and Coventry, 2005). Strack and Deutsch identified two systems determining social behavior: a reflective system that generates behavioral decisions based on knowledge about facts and values, and an impulsive system eliciting behavior through associative links and motivational orientations (Strack and Deutsch, 2004). The dual-process model of addiction postulates that there is an imbalance between a strong activation of the impulsive system and a relatively weak activation of the reflective processes, which leads to the development and the persistence of addictive behaviors (Boendermaker et al., 2015). This model was applied to behavioral addictions, and especially to gambling, where its relevance has been demonstrated to the understanding of both general gambling behavior and GD (Evans and Coventry, 2005). Interestingly, Brevers and colleagues suggested a development of this model with a combination of three key neural systems leading to engaging in and maintaining gambling: (i) a hyperactive “impulsive” system (fast and automatic, motivation-driven and with no deliberate cognitive control); (ii) a hypoactive “reflective” system (slow and deliberate, providing top-down supervision of behavior, and thoughts); (iii) an interoceptive system (bottom-up translation of somatic signals, at the junction between impulsive and reflective systems) (Brevers and Noël, 2013). The results of the first review indicate that the three systems are largely altered in individuals with GD, despite a lack of alterations in general functioning.

#### Alterations of the impulsive system

Alterations of the impulsive system may lead to learned associations through classical conditioning (Brevers and Noël, 2013), development of cognitive biases on the betting outcomes (Evans and Coventry, 2005) and hypersensitization toward gambling-related cues (Brevers and Noël, 2013). Associative representations may then develop between gambling and positive affects, which may induce orientation (engagement) and maintenance of attention toward gambling-related cues and reactivation of gambling-related schemes of action by gambling-related cues, making it difficult for the gambler to control gambling urges. This area of research has to be developed, as the scarcity of studies on the attentional biases and implicit associations, especially in individuals with GD, does not allow the driving of any formal conclusions (only two studies on attentional biases and none on implicit associations between gambling-related cues and representations in memory). In their review on attentional biases, Molde et al. suggested that findings with respect to GD are generally in accord with those concerning substance users and abusers (Molde et al., 2010). Working on attentional biases and implicit associations may reduce the activation of the impulsive system to the benefit of the reflective system, giving the addicted gambler the best chance of controlling his behavior.

#### Alterations of the reflective system

Alterations of the reflective system, and especially executive functioning, have been studied more. A large part of the research on reflective processes has focused on response-inhibition and decision-making capacities.

##### Alterations in response inhibition

Cognitive and motor inhibition (both of the engagement in an action and suppression of an already-engaged action) have been demonstrated to be altered, but the literature is divergent in some aspects. Indeed, nearly one third of the studies identified, which assessed response inhibition in GD, concluded with negative results (no alterations in individuals with GD). This may have been due to the heterogeneity of the tasks used to assess response inhibition, even if there is a sort of a consensus toward three tasks: a Stroop test for cognitive inhibition, with both the classical task or the addiction variant; GNG paradigms (inhibition of engagement in a motor action), and SS paradigms (suppression of an already-engaged motor action) for motor inhibition. However, there exist many variants of each task, making it difficult to produce homogeneous results. Alterations in response inhibition in individuals with GD are supposed to decrease the higher-order control in the impulsive system, so reinforcing impulses to engage in, or maintain, gambling activity (Brevers and Noël, 2013). Enhancing response inhibition, especially in association with gambling-related cues, should be viewed as an equally relevant goal of gambling treatment as work on attention and implicit association, making it possible to restore the balance between impulsive and reflective systems and so to enhance efficient control over gambling behavior.

##### Alterations of decision-making processes

Studies on decision-making deficits have focused on several processes: delay discounting; use of feedback for future decisions; sensitivity to monetary rewards and punishments; anticipatory markers of risk assessment (which are part of the interoceptive system), and general decision-making capacities in risky and/or ambiguous situations. Whatever the process explored, the literature is relatively unanimous and stable in concluding that impairment is present. Delay discounting impairment is characterized by a lesser ability to defer a reward, especially when the reward is high (Petry, 2001; Dixon et al., 2003; Ledgerwood et al., 2009; Billieux et al., 2012; Kraplin et al., 2014). Individuals with GD have been shown to display altered sensitivities to both rewards and punishments, with an increased sensitivity to rewards (Hewig et al., 2010; Brevers et al., 2014; Lorains et al., 2014a) and a decreased sensitivity to punishments (Sharpe, 2004; Lorains et al., 2014a). Insensitivity to losses have sometimes been found to be less pronounced than hypersensitivity to wins (Hewig et al., 2010), especially insensitivity to near losses (Kreussel et al., 2013). This imbalance between sensitivity to wins and losses could result in difficulty emotionally differentiating (subjective excitement) between wins and losses, especially when based on physiological arousal, and so to take feedback into account for making future decisions (Sharpe, 2004). More specifically, individuals with GD seem to attribute less weight to negative feedback for future decisions (Brand et al., 2005; Goudriaan et al., 2005; Hewig et al., 2010). All of these alterations lead to lower decision-making performances in gambling-like situations, but the performance is preserved in other contexts (Ledgerwood et al., 2009; Fauth-Bühler et al., 2014). Decision-making difficulties can lead both to increased losses and the continuation of gambling activity despite losing.

##### Alterations of other executive functions

Other alterations in executive functioning identified in this review are concept generation and flexibility, albeit with results differing between studies. Impairments in concept generation and/or flexibility may lead to difficulties in associating the outcomes of choices and corresponding feedback. While an individual with no alteration will soon realize the random character of gambling outcomes, an impaired individual may consider that gambling outcomes are not the results of random contingencies and may rather try to explain contingencies with non-valid justifications such as acting in a certain way, wearing certain trousers, throwing the dices more or less strongly, etc. This may induce and exacerbate erroneous thoughts about gambling outcomes and contribute to the maintenance of gambling behavior. Given the link between flexibility, concept generation and inhibition (for example, switching from one set to another in flexibility tasks depends on the inhibition of the previous pertinent set), it is likely that it is ultimately impairments in inhibition that indirectly influence performances on flexibility and/or concept generation tasks, which may explain the variation in the results in the literature. Future studies should, therefore, focus on the identification of such individual associations, and their relationship with performance.

##### Alterations of metacognition

Another part of the reflective system is concerned with the metacognitive judgment of decisions. While performing poorly on decision-making tasks, individuals with GD constantly showed higher overconfidence on (wrong) choices (Goodie, 2005; Brevers et al., 2013). As argued by Brevers and Noël (2013), poor decision-making capacities can be driven by poor metacognition. Altered sensitivities to both rewards and losses (Sharpe, 2004; Goudriaan et al., 2006b; Hewig et al., 2010; Brevers et al., 2014; Lorains et al., 2014a) may represent poor monitoring abilities. This may induce a reduction of the flow of information toward the metacognitive library of strategies (Nelson and Narens, 1990), leading in turn to poor adjustment of the cognitive processes involved in the action (attention mobilization, switching of strategies, inhibition of the action, etc.). The lowered ability to take feedback into account, especially negative feedback (loss), for future decisions (Brand et al., 2005; Goudriaan et al., 2005; Hewig et al., 2010) may provide a good illustration of this impaired metacognition. This part of the research on GD is very poor, as there have only been two studies, one conducted in 2005 (Goodie, 2005) and one in 2013 (Brevers et al., 2013). However, it could be of interest to explore the metacognitive capacities in individuals with GD, as this may show up the self-perception of the inability to control behavior and so a lack of motivation to stop it. Therapeutic work on metacognitive capacities could thus be based on overconfidence in terms of making bad choices and the perceived inability to stop gambling.

#### Alterations of the interoceptive system

At the frontier between the impulsive and reflective systems, the interoceptive system can both exacerbate the activation of the impulsive system and undermine the control of the reflective system (Brevers and Noël, 2013). Except studies conducted to identify the brain regions (mainly the insula) activated in cue reactivity (Limbrick-Oldfield et al., 2017), research in this area is very poorly represented. However, physiological arousal in response to gambling may be experienced subjectively as urges (Brevers and Noël, 2013) and these induce implicit associations between certain physiological reactions to gambling-related cues and cravings. For example, Goudriaan et al. found that individuals with GD had decreased heart rates after both wins and losses, while healthy controls had an increase after wins and a decrease after losses (Goudriaan et al., 2006b). This may reinforce the difficulties in monitoring gambling contingencies and thus to adjust behavior accordingly. Sharpe et al. also concluded that there was higher cue reactivity in individuals with GD, which could be limited by using competing thoughts (Sharpe et al., 1995). The interoceptive system may also be involved in anticipatory somatic markers of risk assessment. Indeed, somatic reactions have been observed at an early stage of risky decision-making, i.e., during the few seconds before making a risky choice. These reactions have been found to be altered in individuals with GD, who showed lower anticipatory skin conductance levels and heart rate decreases for disadvantageous choices compared to healthy controls (Goudriaan et al., 2006b). Also, alpha-amylase levels decreased with disadvantageous choices for individuals with GD, but not for controls. Restoring the balance of the interoceptive system in therapy can be beneficial for both reducing its influence on the impulsive and reflective systems, and for diminishing urges for gambling and cravings, which are thought to represent important factors in persistence and relapse (Cornil and Billieux, 2014).

### Synthesis and therapeutic propositions

Taken together, findings suggest that individuals with GD present with several neurocognitive disruptions in all three of the systems involved in addition (impulsive, reflective, and interoceptive). These could represent one of the mechanisms underlying the development and persistence of GD (Romanczuk-Seiferth et al., 2014) and of treatment failures (Alvarez-Moya et al., 2011). The reference treatment for the management of GD for several decades now has been CBT (Korn and Shaffer, 2004; Gooding and Tarrier, 2009; Stea and Hodgins, 2011). Although efficient, at least in the short term, this kind of therapy has several limitations: (i) it does not directly target the endophenotypic neurocognitive processes underlying the addictive vulnerability of the patient, which have been demonstrated to predict relapse in the long term (Goudriaan et al., 2008); (ii) it does not allow work on “hot” emotional states, especially those driven by somatic arousal, which could largely impact on decision-making (Brevers and Noël, 2013); (iii) it is insufficient for a non-negligible proportion of patients in terms of reducing high levels of impulsivity, whereas it is an important target for the prognosis of treatment outcomes in addictive disorders (Boog et al., 2014; Stevens et al., 2014). As a consequence, CR interventions represent a novel and promising approach to gambling addiction care.

According to the dual process model of addiction (Evans and Coventry, 2005; Vandermeeren and Hebbrecht, 2012), gambling addiction is the result of a disturbed balance between impulsive and reflective processes, with strong automatic processes producing continuous impulses to gamble (“bottom up” processes) and low executive control being less effective in regulating them (“top down” processes). CR interventions should be focused on both impulsive (attentional biases and implicit associations) and reflective (executive functioning, especially response inhibition, flexibility and decision-making, and metacognitive judgement) neurocognitive alterations. By working on both impulsive and reflective processes, CR interventions may restore the balance between automatic and controlling levels, allowing the patient to regain control over behavior. Moreover, it is assumed that CR interventions do not only impact this balance, but also allow for an improvement in cognitive restructuring by mobilizing the necessary cognitive resources, and can generalize to non-cognitive aspects. For example, in a study on alcohol-dependent patients, it was found that CR interventions were effective in improving cognitive impairments, and also that the benefits generalized to non-cognitive outcomes such as psychological well-being or cravings (Rupp et al., 2012). Further, interventions aimed at improving patients' cognitive functioning could increase the efficiency of well-established CBT, thus helping to prevent relapses (Pedrero-Perez et al., 2011). CR interventions should be carried out with CBT to improve efficacy. Indeed, using a combination of therapeutic methods adapted to a patient's specific clinical and cognitive needs, especially when CBT is insufficient, will allow practitioners to act on overall functioning, and so to improve the chances of reducing the symptoms of gambling. Biofeedback to complement CR interventions by acting on the interoceptive system should be considered a particularly relevant therapeutic add-on to both CBT and CR interventions.

To date, such validated CR programs do not exist in the field of GD and so have not been tested. We reporting on several CR techniques that may be useful for the treatment of GD, especially with respect to cognitive alterations identified in the first literature review, and to draw a parallel with an addictive disorder for which CR interventions have been studied to a greater extent: alcohol-use disorder.

#### What can be learnt from studies focusing on CR interventions in patients suffering from alcohol use disorder?

Analyzing studies on use of CR interventions in disorders sharing common symptoms (particularly impulsivity and attentional deficit) with GD, such as alcohol-use disorder, is an important approach to establishing research into CR interventions in GD. Indeed, several CR interventions have produced evidence for its efficacy for SUDs [NEuro COgnitive REhabilitation for Disease of Addiction (NECOREDA) program for drug addictions (Rezapour et al., 2015); Cognitive Bias Modification for SUDs (Boendermaker et al., 2015)] or other addictive-like disorders (Cognitive Bias Modification for excessive multiplayer online gamers; Rabinovitz and Nagar, 2015), which suggest a utility of such interventions for all addictive disorders. Note that the comparison with alcohol-dependence is relevant, but, unlike an addiction without substance, some portions of the deficits are associated with chronic, heavy alcohol use, which may arise from the neurotoxic effects of alcohol itself. Restoration of lost cognitive abilities using practice or functional recovery, which exploits undamaged abilities and environmental aids, are the two approaches used in alcohol dependence (Bates et al., 2002).

There is extensive evidence in the literature for cognitive deficits associated with drug use and the efficacy of CR interventions. A recent review by Rezapour et al. reported on 13 clinical trials conducted between 1994 and 2012 (seven on alcohol dependence, five on polysubstances and one on stimulants) and 9 registered clinical trials, which were ongoing, on neurocognitive rehabilitation (three on cocaine, one on heroin, two on alcohol, one on nicotine, one on polysubstances, one unreported) (Rezapour et al., 2016). It was concluded that the use of CR interventions for addictive disorders was promising both in terms of cognitive functions (particularly attention and memory) and outcomes of addiction treatment, with respect to adherence and retention. However, broad variation in the parameters of studies was noted, such as the study's time period, CR tools and methods used (restorative methods vs. strategy/compensatory-based approaches), durations and settings for treatments (inpatient vs. outpatient, individual vs. group session). Such heterogeneity reflects a lack of appropriate and standard protocols and guidelines for CR interventions for addictive disorders. Hence, there are many challenges before CR interventions can be implemented in the treatment of addictions.

Most of the CR interventions in patients suffering from alcohol-use disorder have consisted of attention bias modification (Molde et al., 2010) and approach bias retraining. Supplementary Table 3 summarizes the main CR programs used in alcohol-use disorder, including a comparison with the only program found for GD (Playmancer). Most of the programs used were computerized. However, most of the studies suffer from using small samples, the lack of long-term measures and the lack of an appropriate control group, which limits us to drawing only very general conclusions. Moreover, the targeted populations were highly heterogeneous, from inpatient to outpatient, and alcohol-dependent patients to abstinent alcoholics. Furthermore, the number, duration and frequency of sessions were also greatly heterogeneous, and follow-up assessments were not systematic and short when they were carried out (1–12 months). To the best of our knowledge, there have been no group session programs, only individual ones. Most of the programs were associated with another current treatment, usually CBT, but there was no consensus with respect to when CR intervention occurred (before or during CBT, for example). The goal of each intervention was consistent between programs: retraining single or multiple functions in order to improve outcomes of the treatment of alcohol dependence in terms of recovering and relapse.

#### Application to GD: training the impulsive system

A specific form of CR intervention named cognitive bias modification (CBM) specifically targets automatic processes and has produced promising results in the treatment of addiction (Sofuoglu et al., 2013). CBM has been defined as the “direct manipulation of a target cognitive bias, by extended exposure to task contingencies that favor predetermined patterns of processing selectivity” (Cristea et al., 2015). In a recent meta-analysis, it was remarked that there had been an “exponential growth in the research employing these CBM procedures,” especially in recent years (Cristea et al., 2015).

According to Schoenmakers et al. selective attention training via ecologically validated stimuli may lead to reduced attentional bias toward drug-related cues in the real environment, which may be translated into significant effects in treatment outcomes (real-life applications) (Schoenmakers et al., 2010). These authors identified three factors that appear to increase effectiveness of CBM interventions, based on the literature: (i) motivating the participants; (ii) the presentation of a large number of different stimuli in training; (iii) the performance of multiple training sessions.

Research on CBM has focused mainly on two types of interventions: attention bias modification (ABM) and interpretative cognitive bias modification (CBM-I) (Macleod, 2012; Cristea et al., 2015).

The principle of ABM involves teaching participants to avoid the addiction-related stimuli (usually pictures or words) by directing their attention, without their knowledge, to neutral or other relevant stimuli (Cristea et al., 2015). According to Posner's work on attention, it can be decomposed in several stages: the orientation of attention toward a relevant stimulus, and the disengagement of attention from non-relevant stimuli before the re-orientation of attention toward a relevant stimulus (Douilliez and Philippot, 2008). In a subthreshold sample of problem gamblers (scoring ≥ 3 at the SOGS), Brevers et al. found an effect of gambling-related stimuli on both the orientation (faster detection of gambling-related stimuli) and the disengagement (slower shifting of attention from gambling-related stimuli) of attention (Brevers et al., 2011). Such training programs are usually performed based on the Visual Probe Task, and have demonstrated efficiency (Lopes et al., 2015).

The principle of CBM-I is similar, but focuses on training participants to consistently interpret complex information, such as ambiguous sentences, in a particular direction, either positively or negatively, and more rarely neutrally (Cristea et al., 2015). CBM-I is frequently used in anxiety and depression but has not yet been applied to addiction (Wiers et al., 2013).

CBM also included other interventions, such as concreteness training or approach and avoidance training (Cristea et al., 2015). In the latter, participants are instructed to respond with an approach movement (for example, pulling a joystick that increases the size of a picture) to certain stimuli and respond with an avoidance movement (for example, pushing a joystick that decreases the size of a picture) to others (Wiers et al., 2011). This zooming effect generates a feeling of approach or avoidance toward the associated stimulus, respectively (Wiers et al., 2011).

#### Application to GD: training the reflective system

Controlling processes are usually trained by using either cognitive tasks used for the assessment of the related cognitive function (such as Go No Go, Stop Signal Task, Tower of London, etc.) or exercises that put the patient in a supposed ecological situation.

The large part of addictive-related inhibition training is based on motor inhibition training. Training programs are mainly based on GNG or SS paradigms, the purpose of such training being to increase self-control toward addiction-related items (Turton et al., 2016). The principle of motor inhibition training is to enhance the inhibition of addiction-related cues embedded in those paradigms (Benikos et al., 2013), by associating no-go or the stop signal with addiction-related cues. In studies on motor inhibition training it has been reported that there are both direct effects on inhibitory performance and indirect effects on alcohol or food consumption (Benikos et al., 2013). However, training programs must take into account several parameters, such as the difficulty of a task, which can be manipulated by reaction time deadlines (Benikos et al., 2013), number of sessions or the cues to be used.

Training inhibition can indirectly influence other reflective processes, such as flexibility or decision-making. Interestingly, in a recent study on a gambling sample without GD (Stevens et al., 2015) it was highlighted that the presence of stop signals in gambling decision-making tasks influences gambling by reducing approach behavior and altering the motivational value of the gambling outcome. This is one of the arguments for the transferability of inhibition training to gambling situations, which could have an influence on overall gambling behavior in real-life. As such, training the reflective system should focus on training of the inhibition of gambling-related cues.

#### Application to GD: training the interoceptive system

Physiological arousal in response to gambling-related cues has been proposed to be experienced subjectively as feelings of urges (Brevers and Noël, 2013) and so to have the ability to induce cravings. This phenomenon could be reeducated using biofeedback intervention, which consists of getting the patient to visualize his physiological response to certain stimuli, in order to help him to develop voluntary control over his body, especially in situations that pose the risk of excessive gambling. This could improve control over urges for gambling. Biofeedback interventions offer a promising therapeutic route in psychiatric/psychological care (Canadian Agency for Drugs and Technologies in Health, 2014), and could potentially be successfully applied to gambling addiction treatments. A complementary approach would be to combine CBT and biofeedback, especially in order to associate distractive or competing thoughts and biofeedback. Indeed, the work of Sharpe had demonstrated that the increased physiological arousal in response to gambling-related cues was limited when a cognitive distraction task was added, especially for individuals with GD (Sharpe et al., 1995). As a consequence, training a gambler to exercise voluntary control over his physiological reactions in gambling situations by combining visualization of his reactions with relaxation exercises or mindfulness, and the use of distractive thoughts, could reduce cravings and lead to reduction or arresting of gambling behavior.

Several studies in the literature have focused on mindfulness training in gambling (de Lisle et al., 2012; Shonin et al., 2013; Garland et al., 2014). Garland et al. suggested that mindfulness training can modify neuroplasticity and so target the risk chain of addiction at the attention-appraisal-emotion interface (Garland et al., 2014). de Lisle et al. in their review, proposed a model of relationships between mindfulness, mechanisms of action, and problematic gambling behavior (de Lisle et al., 2012). However, the paucity of research prevents any demonstration of the clear efficacy of mindfulness-based interventions for GD. However, mindfulness training could be relevant if incorporated into GD treatments such as CBT or biofeedback.

### Recommendations for future clinical studies on the use of CR interventions for GD management

Initially, research on certain potential targets for CR interventions in GD should be developed, and especially on clinical samples. Studies need to focus on attentional biases and implicit associations, cue reactivity, and metacognition. Some specific issues, such as a possible association between craving and attentional bias in GD, have been identified and should be investigated in greater depth (Hønsi et al., 2013). Research on social cognition, with only one exploratory study extracted from the present review (Kornreich et al., 2016), must also be developed. Indeed, impaired social cognition can induce difficulties in terms of interacting with others, indirectly inducing or reinforcing social isolation, which is a factor in the initiation and maintenance of gambling behavior.

Secondly, as the assessment tools used have been found to be heterogeneous, the development of a standardized assessment battery for GD is required. This could serve both to provide more relevant results from neurocognitive studies (with several samples assessed using the same battery), to assess the specific cognitive impairments of each patient in order to adapt the CR intervention accordingly (personalized medicine) and to confirm the benefits for the patient on the trained capacities throughout the intervention (patient-centered approach). This could also provide support for the development of a specific training program. Future research must, therefore, focus on determining which tools are best for measuring neurocognitive impairments in relation to GD, which are those that are optimal for re-training them, and how to adapt both the assessment and training tools to each individual (with personalized cues for example).

Research on CR interventions for GD management is desirable, according to previous research on substance-related addictive disorders. For example, the extensive review on the efficacy of CR interventions for substance-related addictive disorders (Rezapour et al., 2016) highlighted that: (i) only a few studies included follow-up assessments and so controlled studies using long-term follow-up should be done in order to explore longer-term outcomes; (ii) earlier studies reported using “paper-and-pencil” for cognitive training, while more recent studies have mainly used computers to deliver intervention; (iii) most studies have applied programs that include a range of cognitive domains; (iv) the exact cut-off point of cognitive performance still potentially benefit from CR interventions remains unknown (some studies were conducted with “cognitively-impaired” patients, whereas some of them took into account patients without notable cognitive impairments and also found a positive effect of CR interventions); (v) some parameters of CR interventions are still unknown such as duration, intensity, frequency of treatment, preferred setting, individual vs. group; (vi) the efficacy of computer-based vs. “paper and pencil” training approaches has not been directly compared in the context of addiction treatment; (vii) further research is needed regarding single vs. multiple targets. These recommendations could be equally applied to future studies on CR interventions for GD management, which have to include follow-up assessments, to use appropriate control groups, to investigate the optimum mode for delivering interventions (paper-and-pencil or computer-assisted), to explore whether programs should focus on single vs. multiple cognitive domains and to determine the breaking point below which a CR intervention will not be beneficial for the patient. Optimal parameters for CR interventions to reach higher efficacies should also be defined: duration (number of sessions, duration of a single session), intensity (increased difficulty of tasks, frequency of sessions), and modality (individual vs. group, home exercises).

Furthermore, CR interventions must be implemented in combination with usual treatment, i.e., CBT. They can also be combined with other approaches such as biofeedback, to improve the global efficacy of treatment using synergistic actions (holistic approach). Virtual reality, for which there is demonstrated evidence of efficacy for the management of GD (Giroux et al., 2013), may provide another route for the improvement of such interventions. Rezapour et al. suggest using a short contract, which include the patient's own goals, for facilitating behavioral changes and also to provide reinforcement for positive behaviors (Rezapour et al., 2016).

CR interventions should also be tailored to individual needs in order to gain more potent effects. Personalized cues should be used as often as possible, specific modules of training should be selected according to the specific impairments of the patient and level of difficulty of baseline exercises, with gradually increases in the level of difficulty adjusted to the patient's level so as to achieve optimal patient performance over CR sessions (Eack, 2012).

Finally, working on treatment adherence is crucially important when dealing with patients suffering from addictive disorders, who usually show low adherence and high drop-out rates (Rezapour et al., 2016). As CR interventions usually require repetitive training and include home exercises, they could be perceived as really boring and restrictive for patients. Serious video-games, such as Playmancer (Tarrega et al., 2015), represent an innovative and promising way forward in providing CR interventions. They allow the motivation and encouragement of patients within a ludic training framework, and the combination of virtual reality, biofeedback, CR interventions and CBT within the same tool.

## Conclusion

GD is a significant public health issue (The Lancet, 2017). Due to the long-term failure of interventions for GD, there is a need to develop novel and innovative approaches that enhance current treatments (Raman et al., 2014). Thanks to recent research in neurocognitive functioning in GD, neurocognitive impairments have been highlighted in motivational (impulsive), controlled (reflective), and physiological (interoceptive) processes, which provide possible targets for novel CR interventions, such as retraining programs. Such novel therapies may be associated with commonly used interventions (such as CBT, educational and motivational interventions) in order to make therapeutic interventions more effective, longer-lasting, and decreasing the risk of relapse.

Given that CR interventions are a relatively novel therapeutic approach to addictions and that there is currently a scarcity of studies, in the literature, on clinical populations suffering from GD, further research is needed to examine the potential targets of such interventions and the effectiveness of different training approaches. The characteristics of a patient who could benefit from CR interventions are still unknown, particularly concerning neurocognitive deficits (which cut-off point?). So far, no consensus has been reached on the optimal parameters for CR interventions: duration, intensity, frequency of treatment, group vs. individual, single vs. multiple cognitive targets, pencil-and-paper vs. computerized delivery, optimal monitoring sessions, feedback type, measuring outcomes, etc. Even though no firm conclusions can be drawn, CR interventions represent a promising adjunct treatment for GD treatment. Rigorously designed studies with appropriate control groups and longer term follow-ups need to be implemented in future studies. This may lead to the development of interventions that could be of value to individuals with GD.

## Author contributions

GC-B and MG-B are the heads of the IGNACE group and obtained the MRSEI grant from the ANR. GC-B and MB conducted the literature research. GC-B and MG-B screened all the studies for eligibility in the first review and GC-B and MB for the second review. GC-B read and reported data from all the studies included and wrote the first draft of the manuscript. MG-B, MB, and CV-V gave feedback on and made adjustments to this draft. All authors, including the IGNACE group, read and approved the final manuscript. This review was conducted at the initiative of and coordinated by the Clinical Investigation Unit “Behavioral Addictions/Complex Affective Disorders” of the University Hospital of Nantes.

### Conflict of interest statement

GC-B and MG-B declare that the University Hospital of Nantes has received funding from gambling industry (FDJ and PMU) in the form of a sponsorship supporting the gambling section of the Clinical Investigation Unit “Behavioral Addictions/Complex Affective Disorders”. Scientific independence toward gambling industry operators is warranted. There were no constraints on publishing. The other authors declare that the research was conducted in the absence of any commercial or financial relationships that could be construed as a potential conflict of interest.

Members of the IGNACE group:

LC is the Director of the Centre for Gambling Research at UBC, which was funded by the Province of BC government and the British Columbia Lottery Corporation. He has received honoraria and travel expenses from Svenska Spel and the National Center for Responsible Gaming, and paid consultancy for Cambridge Cognition.

PG received research grants, during the last 5 years, from Eli Lilly and Servier, and fees for presentations at congresses or participation in scientific boards from Alcediag-Alcen, AstraZeneca, Biocodex, Bristol-Myers-Squibb, Janssen, Lilly, Lundbeck, Naurex, Otsuka, Roche, Sanofi Pasteur MSD, Servier.

AB declares that he has no conflicts of interest for this manuscript. For the period 2012–2017, AB has conducted research funded directly by the Australian or international government, or government-related funding agencies, and industry operators. These include Gambling Research Exchange Ontario, ClubsNSW, Dooleys Club Lidcombe, Aristocrat Leisure Industries, Gaming Technologies Association, Gambling Research Australia, Sportsbet, NSW Department of Trade and Investment (NSW Office of Liquor, Gaming and Racing), La Loterie Romande (Switzerland), Camelot (United Kingdom), La Française des Jeux (France), Loto-Quebec (Canada), and National Lottery (Belgium), and the National Association for Gambling Studies. He has received honorariums from Manitoba Gambling Research Program and GambleAware (formerly UK Responsible Gambling Trust) for grant reviews, and royalties from several publishers for books and book chapters. He has also received travel and accommodation expenses from Svenska Spel (Sweden), Casino Austria, Gambling Research Exchange Ontario, USA National Council on Problem Gambling, Le Comité d'organization Congrès international sur les troubles addictifs, Victorian Responsible Gambling Foundation, North American Association of State and Provincial Lotteries, New Horizons (British Columbia Lottery Corporation), and National Council on Problem Gambling (Singapore) to attend conferences and meetings. All professional dealings have been conducted with the aim of enhancing responsible gambling and harm minimization policies and practices, training counselors in the treatment interventions, and advancing our understanding of the psychology of gambling.

JBH, PT, JCD, AG, MF, RvdB, JB, SA, SJM, LR and IG declare that they have no conflicts of interest.
