# Supplementary material for: Cognitive Remediation Interventions for Gambling Disorder: A Systematic Review
Source: Front Psychol. 2017 Dec 4;8:1961. doi: 10.3389/fpsyg.2017.01961 (PMC5723090; doi:10.3389/fpsyg.2017.01961)
Supplement: Supplementary file 1 [file DataSheet1.docx]

**Tables and figures**

**Table 1 - List and methods of included studies in the first review on potential neurocognitive targets of Cognitive Remediation (CR) interventions for Gambling Disorder (GD) management (n= 49)**

| **Study id** | **Sample size and participants** | **Mean age** | **Main exclusion criteria** | **Objectives** | **Design** | **Neurocognitive measures used*** | **Main limits** |
| --- | --- | --- | --- | --- | --- | --- | --- |
| Sharpe et al, 1995 (45) | 3 groups :  - 13 PG seeking treatment  - 12 high-frequency non-problem gamblers  - 13 low-frequency non-problem gamblers | PG: 37.2  Non-PG high: 33.8  Non-PG low: 37.3 | - psychosis  - addictive disorders | To examine elicitation of arousal by gambling-related cues in PG | Cross-sectional | Five cue-reactivity tasks combined with SCL, frontalis EMG and EKG measures:   - Neutral (reciting the alphabet) - Horse-race video - Poker machine video - Poker machine video with a cognitive distraction task - Imaginal poker machine winning situation | - small sample size  - the cognitive distraction does not allow for identifying the nature of cognitions which influenced autonomic arousal  - laboratory setting  *Additional limitations (not mentioned):*  - only poker machine players |
| Petry, 2001 (84) | 3 groups:  - 21 PG with substance use disorders (SUD)  - 39 PG without SUD  - 26 HC  Matching for education, race, age and gender | PG-SUD: 43.0  PG-no SUD: 44.0  HC: 39.0 | Not reported | To examine discounting rates in PG with and without substance use problems | Cross-sectional | - Delay-discounting procedure | Not reported  *Additional limitations (not mentioned):*  - small sample size for certain groups |
| Cavedini et al, 2002 (50) | 2 groups :  - 20 PG seeking treatment  - 60 HC | PG: 38.5  HC: 30.3 | - somatic illness  For HC :  - Axis I lifetime diagnoses, except nicotine dependence | To evaluate decision-making abilities in PG | Cross-sectional | - IGT  - Weigl’s Sorting Test (WST)  - WSCT | - small sample size for the PG group  - heterogeneous sample  - lack of homogeneity on some variables  - single measure of decision-making |
| Alessi and Petry, 2003 (115) | 62 PG in treatment | 44.0 | - non-English speaking  - uncontrolled major psychosis  - active suividality | To examine the relationship between severity of PG and impulsivity, using a delay discounting of monetary rewards procedure | Cross-sectional | - Delay Discounting Task | - monetary rewards were hypothetical  - only one monetary reward magnitude was examined |
| Dixon et al, 2003 (82) | 2 groups :  - 20 PrG  - 20 HC  Matching for sex , age, income and level of education | PrG: 40.6  HC: 40.0 | Nor reported | To determine if PG discount delayed monetary rewards to a greater degree than HC | Cross-sectional | - Hypothetical choice task for delay discounting assessment | - many uncontrolled variables as the study was conducted in naturalistic settings  - verbal report of discounting rather than observable behavior  *Additional limitations (not mentioned):*  - identification of PrG with the SOGS (≥ 5) |
| Potenza et al, 2003 (59) | 2 groups :  - 13 male PG  - 11 male HC | PG: 35.2  HC: 29.0 | - age under 18 or over 65  - major neurological injury or illness | To test attention and response inhibition during the presentation of congruent and incongruent stimuli in PG, and to identify the neural correlates | Cross-sectional | - Stroop task coupled with fMRI | -small sample size  - only males  - no matching between PG and HC |
| Sharpe, 2004 (86) | 2 groups :  - 13 PG seeking treatment  - 20 HC | PG: 37.2  HC: 36.8 | - addictive disorders  - psychotic illness | To examine autonomic arousal in response to imaginal win or lose situations | Cross-sectional | - Imaginal “win” or “lose” tasks combined with SCL measure | - small sample size  - only poker machine players seeking treatment for the PG group  - laboratory setting and imaginal tasks  *Additional limitations (not mentioned):*  - recruitment of HC from staff only |
| Brand et al, 2005 (88) | 2 groups :  - 25 male PG in treatment  - 25 male HC  Matching for age, education and intelligence | PG: 40.1  HC: 40.7 | - neurological or psychiatric symptoms | To identify decision-making deficits in PG patients, and to correlate them with executive functioning | Cross-sectional | - M-WSCT  - Stroop  - Word Color Interference Test  - DemTect  - Game of Dice Task | Not reported  *Additional limitations (not mentioned):*  - small sample size  - only males |
| Goodie, 2005 (48) | STUDY 1 - 3 groups:  - 32 PrG  - 34 at-risk gamblers  - 134 non-PrG controls  STUDY 2 - 3 groups:  - 89 PrG  - 68 at-risk gamblers  - 227 non-PrG controls | Not reported | Not reported | To compare JPr and non-PrG controls on decision-making under uncertainty (outside of a gambling context), and to examine the impact of perceived control | Cross-sectional  2 studies | STUDY 1  - Betting-on-confidence task (bet on the participants’ answer)  STUDY 2  - Betting-on-confidence task (bet on the participants’ answer or on a random event) | - range restriction of demographic variables  - features different of gambling modalities  - lack of ecological validity  - definition of control  *Additional limitations (not mentioned):*  - identification of PrG and at-risk gamblers with the SOGS (≥ 5 / 3-4)  - only students |
| Goudriaan et al, 2005 (51) | 4 groups:  - 48 PG in treatment  - 46 abstinent Alcohol dependence (AD) patients in treatment  - 47 Tourette syndrome (TS) patients in treatment or not  - 49 HC  Matching for age, gender and intelligence | PG: 39.0  AD: 47.7  TS: 37.0  HC: 35.8 | - substance abuse or dependence (except alcohol for the AD group)  - severe cognitive impairment  - major psychiatric disorders  - current treatment for mental disorders other than those studied  - certain physical conditions  - use of psychotropic medications  - positive urine screen for alcohol, cannabis or benzodiazepines | To examine whether the decision-making profile of PG resembles more to an impulse control disorder or a substance dependence disorder | Cross-sectional | - IGT  - Card Playing Task  - Go / No-Go discrimination task | - only PG in treatment  - exclusion of comorbid psychiatric disorders  - tasks selected not allowing a comprehensive survey on decision-making |
| Goudriaan et al, 2006 (52) | 2 groups:  - 46 PG in treatment  - 47 HC | PG: 37.8  HC: 35.9 | - alcohol or substance use disorder  - major psychiatric disorders  - current treatment for mental disorders other than PG  - certain physical conditions  - use of psychotropic medications  - positive urine screen for alcohol, cannabis or benzodiazepines  For HC:  - treatment for a mental disorder in the past 3 years | (1) To assess the role of somatic markers in the behavioral performance of PG on the IGT  (2) To assess the physiological reactions after experiencing wins and losses in PG and HC  (3) To examined whether reward and punishment sensitivity could explain differences in physiological reactions between PG and HC | Cross-sectional | - Decision-making: IGT  - Psychophysiological measures: EKG and SCL | - predominance of males  - no real losses of private money (only a proportion of wins) => ecological validity  - exclusion of PG with comorbid alcohol or substance dependence |
| Goudriaan et al, 2006 (34) | 4 groups:  - 49 PG in treatment  - 48 abstinent Alcohol dependence (AD) patients in treatment  - 46 Tourette syndrome (TS) patients in treatment or not  - 50 HC  Matching for age, gender and intelligence | PG: 37.3  AD: 47.2  TS: 36.8  HC: 35.6 | - substance abuse or dependence (except alcohol for the AD group)  - major psychiatric disorders  - current treatment for mental disorders other than those studied  - certain physical conditions  - use of psychotropic medications  - age aver 60 and under 18  - positive urine screen for alcohol, cannabis or benzodiazepines | (1) To investigate whether EFs (executive functions) are impaired in PG compared to HC  (2) To assess the specificity of EF deficits in PG compared to abstinent AD and TS  (3) To assess the role of comorbid psychopathological conditions in the performance of EF tasks | Cross-sectional | EFs:  *- Inhibition*: SST, Circle Tracing Task, Stroop Colour-Word Test  *- Time estimation*: Time estimation and reproduction tests  *- Cognitive flexibility*: WCST, COWAT  *- Working memory*: Self-Ordered Pointing Task-abstract designs, WAIS Digit Span Forward and Backward  *- Planning*: Tower of London  Basic cognitive functions:  - SST mean reaction time  - WAIS Digit Span Forwards  - Benton Visual Retention Test  - Sorting task of the Groningen Intelligence Test | - predominance of male  - only adults  - comorbid psychopathological conditions not very frequent to respond to the third hypothesis  - selection bias due to the matching of AD and TS groups to the PG group  - only PG in treatment  - cross-sectional design (no causal conclusions) |
| Fuentes et al, 2006 (62) | 2 groups:  - 162 PG in treatment with comorbidity (PGC)  - 52 PG in treatment without comorbidity (PGWC)  - 82 HC | PGC: 42.7  PGWC: 40.1  HC: 40.9 | - clinical impairment of sensory, motor, and high cortical functions  For HC :  - any current psychiatric disorder  - recurrent psychiatric syndrome | (1) To examine if performance at Go/No-Go tasks can discriminate between PG and HC  (2) To examine if performance at Go/No-Go tasks is related to self-reported impulsivity | Cross-sectional | - Simple Choice Auditory and Visual Reaction tasks (Go/No-Go tasks) | - cross-sectional design  - no assessment of ADHD |
| Kertzman et al, 2006 (116) | 2 groups :  - 62 PG in treatment  - 83 HC | PG: 40.6  HC: 40.4 | For PG only:  - comorbid axis I psychiatric disorders  - neurological disorders  - mental retardation  - any use of alcohol or other substances of abuse in the previous 4 weeks  - treatment with psychiatric medication in the previous month  - on-going psychiatric care | To examine interference control in PG | Cross-sectional | - Stroop - reverse variant | - selection bias (recruitment from an ambulatory setting and exclusion of comorbid addictive disorders)  - results of the Stroop test not correlated with other tests of executive functioning and personality traits |
| Rodriguez-Jimenez et al, 2006 (117) | 3 groups:  - 16 PG in treatment with childhood ADHD  - 39 PG in treatment without childhood ADHD  - 40 HC | PG-ADHD: 31.8  PG-no ADHD: 34.6  HC: 32.0 | - psychotic disorders  - affective disorders  - organic mental disorders  - substance use (except nicotine and caffeine) in the previous year  - certain physical conditions  - illiteracy  - IQ < 70  Only for HC: presence of PG | To investigate the influence of childhood ADHD history in PG, especially impulsivity | Cross-sectional | - SST  - Differential Reinforcement of Low Rate Responding Task  - CPT | - only males  - small sample size for the PG-ADHD group  - retrospective assessment of childhood ADHD  - exclusion of substance use in the previous year |
| Linnet et al, 2006 (70) | 2 groups :  - 61 PrG  - 39 non-problem gamblers | PrG: 35.3  Non-PrG: 26.6 | For non-problem gamblers: SOGS > 3 | To develop quantifiable behavioral measures of chasing in a gambling related decision making situation | Cross-sectional | - Mouse game (modified version of the IGT without monetary rewards) | - modified version of the IGT (non-monetary rewards and no audio feedback)  - only psychology students for the non-problem gamblers group => age and gender differences between groups  *Additional limitations (not mentioned):*  - identification of PrG with the SOGS (≥ 5)  - small sample size for female (sub analyses on gender) |
| Kalechstein et al, 2007 (35) | 3 groups :  - 9 PG  - 29 methanphetamine dependent individuals (MD)  - 19 HC | PG : 53.7  MD : 34.8  HC : 32.5 | - history of stroke, traumatic brain injury, epilepsy, or ADHD  - HIV seropositivity  - abuse or dependence on any drug (except metham-phetamine for MD)  - positive urine toxicology screen (except metham-phetamine for MD)  For HC:  - pathological gambling | To determine whether PG demonstrate frontal lobe impairments on measures used by clinician and in comparison to methamphetamine dependent individuals | Cross-sectional | - Ruff Figural Fluency Test  - Stroop Color-Word Test  - TMT, part B  - National American Reading Test | - PG older than other groups  - small sample size, especially for the PG group  - cross-sectional design |
| Labudda et al, 2007 (118) | 2 groups:  - 22 PG in treatment  - 19 HC | PG: 40.5  HC: 42.9 | - neurological and/or psychiatric disease | To investigate the relationship between decision-making deficits and neuroendocrine reactivity in PG | Cross-sectional | - Game of Dice Task combined with salivary cortisol and alpha-amylase levels | - no direct somatic marker for sympathetic nervous system activity or catecholamine activity assessed  *Additional limitations (not mentioned):*  - small sample size  - only males |
| Lakey et al, 2007 (54) | 3 groups :  - 79 PG  - 85 PrG  - 57 non-problem gamblers  Only frequent card players | 19.2 | Not reported | To examine the links between biased judgment and decision-making in PG | Cross-sectional | - Georgia Gambling Task  - IGT | - only frequent card players  - only students  - no involvement of real monetary reward |
| Leiserson et Pihl, 2007 (64) | 3 groups:  - 14 male PrG  - 24 male at-risk gamblers  - 19 male non-problem gamblers | PrG: 26.4  At-risk: 22.6  Non-PrG: 22.9 | - serious health problems or psychiatric disorder | To explore the underlying mechanisms under perseverative chasing in PrG, especially oversensitivity to reward, deficient inhibition of reward-seeking behavior and working memory deficits | Cross-sectional | Measures of perseveration:  - *impulsivity*: reward-punishment version of the Go/no-go task  - *working memory*: self-ordered pointing task, spatial and non-spatial conditional association tasks | Not reported  *Additional limitations (not mentioned):*  - only males  - small sample size  - identification of PrG with the SOGS (≥ 5) |
| Goudriaan et al, 2008 (40) | 46 PG in treatment, abstinent from gambling for less than 3 months at inclusion:  - 24 relapsers (PGR)  - 22 non-relapsers (PGNR) | PGR: 40.0  PGNR: 36.3 | - substance abuse or dependence  - major psychiatric disorders  - certain physical conditions  - use of psychotropic medications | To investigate the predictive value of self-reported and neurocognitive measures of inhibition and decision-making on relapse in PG, 1 year after treatment | Longitudinal | *- Disinhibition*: SST, Stroop Colour-Word Task  *- Decision-making under conflicting contingencies*: IGT, Card Playing Task | - small sample size  - limited number of predictors studied  - low total variance accounted by the model  - restricted generalization (PG in treatment, without comorbid disorders) |
| Forbush et al, 2008 (41) | 2 groups :  - 25 PG  - 34 HC | PG: 46.9  HC: 41.9 | - convulsive disorder  - primary neurological disorder  - psychotic, cognitive or bipolar disorder  For HC:  - substance abuse  - current Axis I or II psychopathology | To test whether there are important differences in predictive variance of both neuropsychological characteristics and personality traits in PG | Cross-sectional | - Stroop Test  - Letter-Number Sequencing subtest (WAIS)  - TMT  - WCST-64  - IGT  - Picture Completion subtest (WAIS)  - COWAT  - Boston Diagnostic Aphasia Examination Animal Naming Test  - Wide Range Achievement Test-3 Reading Scale | - differences in education level and sex between the two groups  - no examination of Axis I disorders in PG  - self-selected sample  - combination of current and past PG  - small sample size  - many independent variables |
| Kertzman et al, 2008 (63) | 2 groups:  - 83 PrG in treatment  - 84 HC | PrG: 39.5  HC: 36.8 | For PG:  - any ongoing psychiatric treatment  - neurological or major psychiatric disorders  - mental retardation  - alcohol and substance abuse or dependence  - treatment with any psychiatric medication in the previous month  For HC : any current or lifetime psychiatric disorder | To compare performance on two inhibition tasks requiring inverted go event frequency (CPT and go/no-go tasks) in PrG vs HC | Cross-sectional | - *target detection*: CPT  - *response inhibition*: Go/no-go | - cross-sectional design  *Additional limitations (not mentioned):*  - identification of PrG with the SOGS (≥ 5) |
| Marazziti et al, 2008 (119) | 2 groups :  - 20 PG in treatment  - 20 HC | PG: 26.2  HC: 25.3 | Not reported | To explore the brain area related to PG by means of neuropsychological assessment | Cross-sectional | - WSCT  - Wechsler Memory Scale R  - Verbal Associative Fluency Test | - small sample size  - high level of comorbid disorders |
| Roca et al, 2008 (57) | 2 groups:  - 11 PG recruited in ecological settings  - 11 HC | Nor reported | - psychosis or major psychiatric comorbidity  - head injury or seizure disorder | To assess the relationship between decision-making deficits and broader executive ability in PG | Cross-sectional | - IGT  - Go/No-Go | - small sample size |
| Alvarez-Moya et al, 2009 (58) | 3 groups :  - 15 female PG in treatment  - 15 female with Bulimia Nervosa (BN) in treatment  - 15 female HC | PG: 44.4  BN: 33.6  HC: 35.5 | - male  - neurological disorder or head injury, psychotic disorder or comorbidity among PG and BN groups  - substance abuse in the previous 3 months | To explore similarities and differences in executive functioning in female PG and BN patients, relative to HC females | Cross-sectional | - Stroop Color and Word Test  - WSCT | - small sample size  - only females  - no measure of axis I comorbidity |
| Ledgerwood et al, 2009 (66) | 3 groups :  - 31 PG with Substance Use Disorders (SUD)  - 30 PG without SUD  - 41 HC | PG-SUD: 44.5  PG-no SUD: 48.4  HC: 45.7 | - acute and severe psychiatric disorders  - current substance dependence (except nicotine or caffeine)  - positive toxicology screen for any substance | To assess various dimensions of impulsivity in PG, in comparison with SUD | Cross-sectional | - Delayed Discounting of Monetary Rewards task  - Single Key Impulsivity Paradigm  - Immediate and Delayed Memory Tasks  - GoStop Impulsivity Paradigm  - Baloon Analogue Risk Task  - Paced Auditory Serial Addition Task | - presence of a history of SUD rather than current SUD  - small sample size |
| Hewig et al, 2010 (85) | 2 groups:  - 21 male student PG  - 22 male student HC  Matching for age, sex and handedness | PG: 23.0  HC: 23.5 | Not reported | To assess reward-related neural response in PG | Cross-sectional | - Psychophysiological measures: EEG and EOG, combined with a Black Jack simulator | Not reported  *Additional limitations (not mentioned):*  - only students  - small sample size |
| Kertzman et al, 2010 (120) | 2 groups :  - 82 PG in treatment  - 82 HC | PG: 39.2  HC: 39.5 | For PG:  - neurological disorders  - mental retardation  - alcohol and substance abuse / dependence  - major psychiatric disorders  - treatment with psychiatric medication in the previous month  For HC : any current or lifetime psychiatric disorder | To compare performance on the MFFT (decision-making) in PG compared to HC, especially on the speed-accuracy tradeoff | Cross-sectional | - MFFT (computerized variant) with instructions for accuracy (not for speed) | - only seeking-treatment PG  - exclusion of PG with comorbid addictive disorders  *Additional limitations (not mentioned):*  - recruitment of HC among staff members |
| Molde et al, 2010 (81) | 2 groups :  - 36 treatment-seeking PG (only slot-machine gamblers)  - 22 HC  Matching for age and gender | PG: 40.5  HC: 41.2 | - psychosis or organic mental disorder  - concurrent drug or alcohol dependency | To explore and extent the addiction Stroop task for gambling, using both supraliminal and subliminal presentations of pictorial gambling-related stimuli | Cross-sectional | - Stroop task - pictorial variant | - low number of trials in each condition and long inter stimulus interval  - no examination of the Stroop effect due to gambling in general  - majority of HC did not gamble of slot machines (bias in stimuli familiarity)  - only treatment-seeking PG  - exclusion of comorbid addictive disorders  *Additional limitations (not mentioned):*  - only slot-machine gamblers for PG |
| Kertzman et al, 2011 (53) | 2 groups :  - 51 PG seeking treatment  - 57 HC | PG: 39.5  HC: 37.7 | For PG:  - major psychiatric disorders  - alcohol and substance abuse / dependence  - neurological disorders  - mental retardation  - treatment with psychiatric medication in the previous month  For HC : any current or lifetime psychiatric disorder | To examine the extent to which IGT performance in PG is influenced by individual differences in inhibition ability | Cross-sectional | - IGT  - Go - No Go / CPT  - Stroop (reverse variant) | - no examination of effect of comorbid Axis I disorders  - only treatment-seeking PG  - no examination of comorbid Axis II disorders  - selection of measures of assessing impulsivity  *Additional limitations (not mentioned):*  - recruitment of HC among staff members |
| Alvarez-Moya et al, 2011 (92) | 88 consecutive patients seeking treatment for PG  (post-exclusion of patients who have chosen not to start treatment (n=27 over 115)) | 36.7 | - age under 18 or over 65  - neurologic disorder or head injury  - psychotic disorder  - substance abuse in the past 3 months | (1) To establish links between neurocognitive variables and self-report measures of impulsivity in PG  (2) To determine the predictive value of both neurocognitive and self-report measures of impulsivity on treatment outcome (dropout & relapse) during treatment in PG | Cross-sectional | - WSCT  - Stroop color & word test  - TMT  - IGT ABCD-EFGH  - F-A-S test of verbal fluency  - Backward Digits Span task  - Corsi blocks task: Backward Blocks Span | - relapse and dropouts measured during treatment and not after treatment completion – no follow-up data  - no control group  - exclusion of patients who have chosen not to start treatment (who differed from the final sample on impulsivity and executive functioning) => bias toward a “healthier” PG group  - over-representation of slot-machine gamblers |
| Odlaug et al, 2011 (67) | 3 groups :  - 46 PG  - 69 at-risk gamblers  - 135 non-problem gamblers | PG: 45.4  At-risk: 22.5  Non-PG: 23.4 | - current axis I disorders  - brain injury/trauma  - history of seizures  - implementation or dose change of psychoactive medication in the 6 weeks of study enrollment  - inability to understand or consent | To examine clinical features, response inhibition and cognitive flexibility in gamblers with varying clinical severity | Cross-sectional | - SST  - IDED set-shift test | - PG were older than other groups  - PG were mainly treatment-seeking individuals  - no record of IQ  - use of psychoactive medication in a number of subjects |
| Albein-Urios et al, 2012 (33) | 3 groups :  - 29 cocaïne dependent individuals (CDI) starting treatment  - 23 PG starting treatment  - 20 HC  Matching for years of education and full IQ scores | CDI: 33.2  PG: 35.6  HC: 28.6 | - comorbid axis I and II disorders, except alcohol abuse and nicotine dependence  - head injury or diseases affecting the central nervous system  - other treatments within the 2 years before inclusion  - entering treatment by court request | To identify the cognitive processes :  (1) altered in both CDI and PG (common addictive vulnerability)  (2) specifically altered in CDI (neurotoxic effects of cocaine) | Cross-sectional | - Stroop task (D-KEFS / CWIT)  - N-back | - small group sample size  - lack of correlation between PG measures and impulsivity and cognitive indices => more refined measures should be used for PG  - lower cocaine consumption rate in the CDI group compared to other studies |
| Billieux et al, 2012 (68) | 2 groups:  - 20 PG in treatment  - 20 HC  Matching for sex, age and socio-educational level | PG: 44.1  HC: 38.3 | - substance use disorder (except tobacco dependence)  - neurological disorders  For HC:  - past and present gambling practices  - psychiatric or neurological disorders | To determine whether heterogeneous “impulsivity profiles” could be highlighted among PG | Cross-sectional | - Go-stop impulsivity paradigm  - Single Key Impulsivity Paradigm | Not reported  *Additional limitations (not mentioned):*  - small sample size |
| de Ruiter et al, 2012 (65) | 3 groups :  - 19 PG seeking treatment  - 19 heavy smokers (HSM)  - 19 HC | PG: 35.3  HSM: 33.8  HC: 34.7 | - schizophrenia or psychotic episodes  - current manic disorder  - treatment for other mental disorders or by a neurologist  - age under 18 years  - consumption of psychotropic medications or substances  - history of drug or alcohol abuse  - systemic disease, brain trauma or exposure to neurotoxic factors  For HC:  - smoker  - gambling activity > twice a year  For HC and HSM:  - anxiety disorders, depression, OCD, PTSD and ADHD | To investigate whether PG and heavy smokers would show a similar pattern of neural dysfunction during response inhibition | Cross-sectional | - SST coupled with fMRI imaging | - different recruitment strategies for PG and HSM  - smoking history of the HSM not recorded  *Additional limitations (not mentioned):*  - small sample size |
| Grant et al, 2012 (121) | 501 PG (274 females and 227 males)  77 for the neuro-psychological assessment (35 females and 42 males) | Female: 48.7  Male: 45.9 | - psychotic or bipolar disorders  - inability to understand and consent  - current substance abuse or dependence (past 12 months) | To examine clinical and cognitive commonalities and differences in males vs females with PG, especially on  (1) the progression of the disorder  (2) the level of impulsivity | Cross-sectional | *- Cognitive flexibility*: IDED set shift task  *- Motor inhibition*: SST | - uncorrected p-value  - small effect sizes  - data pooled from subjects across several research trials  - small sample size for the neuropsychological assessment, and not all subjects drug-free |
| Grant et al, 2012 (69) | 2 groups:  - 77 treatment-seeking PG (22 strategic and 55 non-strategic)  - 28 HC  Matching for age and gender | PG: 42.7  HC: not reported | - psychotic or bipolar disorders  - current substance abuse or dependence (past 12 months)  - unstable psychotropic medication | To examine clinical and cognitive characteristics (response inhibition and cognitive flexibility) of PG based on preference of gambling activity (strategic vs non-strategic games) | Cross-sectional | *- Cognitive flexibility*: IDED set shift task  *- Motor inhibition*: SST | - collection of the preferred type of gambling but many gamblers played a variety of games  - only treatment-seeking PG  - no historical treatment information  - limited number of cognitive functions explored  - current intake of psychoactive medications for some patients |
| Hur et al, 2012 (42) | 3 groups:  - 16 PG in treatment  - 31 patients with OCD in treatment  - 52 HC  Matching for age, education and IQ | PG: 28.3  OCD: 26.9  HC: 25.1 | - head injury, medical or neurological disorders, or alcohol or drug abuse  - IQ < 80  - age < 19 years | To investigate and compare the neurocognitive profiles of PG and OCD | Cross-sectional | - *IQ*: K-WAIS  - *executive functioning*: TMT, COWAT, category fluency test, Stroop task and WSCT  - *verbal learning and memory*: California Verbal Learning Test (Korean version)  - *visual organization and memory*: Rey-Osterrieth Complex Figure Test | - no female in the PG group (unlike other groups)  *Additional limitations (not mentioned):*  - small sample size of the PG group |
| Ledgerwood et al, 2012 (55) | 2 groups:  - 45 PG  - 45 non-pathological gamblers controls | PG: 46.1  Non-PG: 45.8 | - current and uncontrolled mania, psychosis or suicidality  - substance dependence except nicotine or caffeine  - positive toxicology screen for substances  - positive alcohol breathalyzer  For non-PG only: no lifetime or current PG | To compare PG and non-PG on executive functioning | Cross-sectional | - WSCT  - Stroop task  - COWAT  - tower of London  - IGT  - GoStop Impulsivity paradigm  - Wechsler Memory Scale III  - WAIS | - recruitment of PG from both treatment and community sources  - cross-sectional design  - somewhat small sample size  - no detailed assessment of psychotropic medication use |
| Brevers et al, 2013 (47) | 2 groups :  - 30 PG  - 35 HC | PG:39.9  HC: 44.1 | - current comorbid Axis I disorders  - significant medical illness  - head injury  - use of psychotropic drugs or substances that influence cognition  - overt cognitive dysfunction  For HC:  - Axis I disorders  - drug use disorder in the past year  - consumption of more than 54g/day of alcohol for longer than one month | To explore the nature of the metacognitive capacities involved in decision-making under uncertainty with post-decision wagering in PG | Cross-sectional | - IGT with post-decision wagering  - General neuropsychological functioning: WAIS IQ, Operation-span Task | - post-decision wagering could be influenced by other cognitive aspects  - advantageous wagering could be learned implicitly, without explicit awareness  - no representation of the extreme ends of the spectrum of PG  - IGT performance could be influenced by other cognitive or emotional factors  *Additional limitations (not mentioned):*  - not the same exclusion criteria for HC and PG |
| Kreussel et al, 2013 (87) | 2 groups:  - 20 male PG students  - 21 male HC students  Matching for sex, age and handedness | PG: 23.0  HC: 24.0 | Not reported | To examine the neurophysiologic sensitivity (and time course) to negative feedback (near and full losses) of PG compared to HC | Cross-sectional | - Psychophysiological measures: EEG and EOG, combined with a Black Jack simulator - measure of the event-related potential and behavioral data after negative feedback | - small sample size  *Additional limitations (not mentioned):*  - the PG group had a mean score to the SOGS under the threshold for problem gambling  - only students |
| Ochoa et al, 2013 (122) | 131 PG in treament | 37.2 | - missing value for diagnostic items  - psychiatric illness  - neurological illness | To characterize decision-making deficits in PG and to examine predictors during two types of decisions: under ambiguity and under risk | Cross-sectional | - IGT ABCD-EFGH  - WCST  - Stroop task  - TMT  - Digits Backward Task  - Measure of estimated intelligence: vocabulary subtest of the WAIS-III | - restricted generalization (overrepresentation of slot-machine gamblers, without comorbid disorders)  - lack of a HC group |
| Vizcaino et al, 2013 (43) | 2 groups:  - 23 PG  - 21 HC | PG: 45.8  HC: 39.8 | - abnormal uncorrected vision  - DSM-IV disorder (except nicotine dependence)  - alcohol consumption above 60g/day in men or 40g/day in women  - previous knowledge of attentional tasks | To assess attentional bias at the level of maintenance of attention in PG | Cross-sectional | - Visual Probe Task with exposure times that assess maintenance of attention | - HC were not gamblers  - PG group individuals had only severe PG  - only treatment-seeking PG  - no assessment of the relationship of attentional biases with risk factors for PG |
| Brevers et al, 2014 (49) | 2 groups :  - 20 PrG  - 20 HC | PrG: 36.0  HC: 37.1 | - comorbid substance use disorder (on the basis of the Addiction Severity Index Short Form)  For HC :  - Previous knowledge of the tasks | To investigate more deeply the specific basic cognitive processes that underlie impaired IGT performance in PrG, using the Expectancy-Valence (EV) model | Cross-sectional | - IGT - scored according to the EV model | - small sample size  - identification of PrG with the SOGS (≥ 5) |
| Fauth-Bühler et al, 2014 (71) | 2 groups:  - 80 male PG in treatment  - 89 male HC  The 2 groups were subdivised between low and high depressive states | PG: 37.4  HC: 36.2 | - contraindication to fMRI  - positive urine drug screen  - current use of psychotropic medications  - major physical disorder  - acute psychosis  - inadequate language skills  For HC:  - axis I psychiatric disorder | To assess effort-dependent monetary reward processing in a large sample of PG compared to HC, and to examine the impact of depressive mood state | Cross-sectional | - Effort-dependent instrumental-motivation task without any gambling-like features, coupled with fMRI imaging | - high depressive state defined with a score in a lower range than the usual clinical values, especially for the HC group  - only males |
| Kraplin et al, 2014 (83) | 2 groups:  - 19 male PG  - 19 male HC  Matching for age, gender and smoking status | PG: 31.4  HC: 30.6 | - age under 18  - psychotropic medication in the last three months  - current treatment for mental disorders  - disorders which might influence cognitive or motor performance  - mother tongue other than german  - current other mental disorders, except nicotine dependence | To investigate the relation of impaired decision-making (both motivational and reflective components) and impulsivity in PG | Cross-sectional | - CGT  - Delay Discounting Paradigm | - comorbid ADHD assessed by verbal report only  - Axis II disorders not assessed  - possible neurotoxic effects of life-time substance use disorders  - convenience sample  - small sample size |
| Lorains et al, 2014 (56) | 2 groups:  - 39 PrG in treatment (15 strategic and 24 non-strategic gamblers)  - 41 HC  Matching for gender, age and IQ | PrG: 46.6  HC: 44.3 | - age over 65  - head injury  - neurological disorders  - psychotic disorders  - alcohol or illicit drug use in the previous 12 hours  For HC :  - mental health disorders  - moderate or severe PrG  - gambling more than monthly | To examine the cognitive, motivational and response style processes underlying PrG’s decision-making under condition of risk and ambiguity, and sensitivity to loss aversion, and to compare strategic and non-strategic PrG | Cross-sectional | - IGT with the PVL model for decomposing IGT performance  - loss aversion task | - only treatment-seeking PrG  - comorbidities may have influenced performance  - HC were not gamblers  *Additional limitations (not mentioned):*  - identification of PrG with the CPGI (≥ 8) |
| Lorains et al, 2014 (44) | 2 groups:  - 39 PrG in treatment (15 strategic and 24 non-strategic gamblers)  - 41HC  Matching for gender, age and IQ | PrG: 46.6  HC: 44.3 | - age over 65  - head injury  - neurological disorders  - psychotic disorders  - alcohol or illicit drug use in the previous 12 hours  For HC :  - mental health disorders  - moderate or severe PrG  - gambling more than monthly | (1) To examine self-reported impulsivity and inhibitory control in PG to delineate which of the related construct are associated with PrG  (2) To examine the relationship between self-reported impulsivity and inhibitory control in PrG, especially between strategic and non-strategic PrG | Cross-sectional | - SST  - emotional Stroop task  - Sustained Attention of Response Task  - Random Number Generation task | - overrepresentation of women  - lack of power  - heterogeneity of PrG  - comorbidities may have influenced performance  - motor rather than vocal response in emotional Stroop task  - unrealistic environment  *Additional limitations (not mentioned):*  - identification of PrG with the CPGI (≥ 8) |
| Kornreich et al, 2016 (72) | 2 groups:  - 22 PG in treatment  - 22 HC  Matching for age and education level | PG: 41.5  HC: 40.1 | - bipolar disorder  - schizophrenia  - alcohol or other drugs dependence, except nicotine  - dementia | To investigate whether PG shares deficits of emotional non-verbal signals with substance-use disorders | Cross-sectional | - Three emotion recognition tasks (musical, vocal and facial) | - no consideration of the heterogeneity of PG  - only males  - small sample size  - exclusion of comorbidities which limits the generalization of findings |

* as the review focuses only on the neurocognitive processes altered as part of the endophenotype, only neurocognitive tasks are reported here and self-reported questionnaires or clinical interviews are not mentioned.

Studies are reported in chronological order.

**PG**: pathological gamblers/gambling (diagnosed with DSM or ICD criteria); **PrG**: problem/excessive gamblers/gambling (identified with a SOGS scores ≥ 5 or a CPGI ≥ 8); **HC**: Healthy controls; **OCD**: Obsessive-Compulsive Disorder; **ADHD**: Attention Deficit Hyperactivity Disorder; **PTSD**: post-traumatic stress disorder

**UPPS**: UPPS impulsive behavior scale; **TCI-R**: Temperament and Character Inventory Revised; **SOGS**: South Oaks Gambling Screen, **VAS**: Visual Analog Scale; **CPGI**: Canadian Problem Gambling Index

**WSCT**: Wisconsin Card Sorting Test (Modified version: M-WCST); **TMT**: Trail Making Test; **IGT**: Iowa Gambling Task (ABCD-EFGH version for the assessment of sensitivity to reward and punishment respectively); **CGT**: Cambridge Gambling Task; **COWAT**: Controlled Oral Word Association Test; **CPT**: Continuous Performance Test; **SST**: Stop-Signal Task; **WAIS**: Wechsler Abbreviated Intelligence Scale; **MFFT**: Matching Familiar Figures Test; **IDED set-shift test**: Intra-dimensional/extra-dimensional set-shift test

**EKG**: Electrocardiogram; **SCL**: Skin Conductance Level; **EEG**: Electroencephalogram; **EOG**: Electro-oculogram; **EMG**: Electromyogram

**Table 2 - Synthesis of the main results about neurocognitive processes altered in Gambling Disorder (GD)**

| **Type of cognitive function assessed** | **Study id*** | **Measure(s) used** | **Main results** |
| --- | --- | --- | --- |
|  |  |  |  |
| **GENERAL COGNITIVE FUNCTIONING** |  |  |  |
| **Speed of information processing** | Brand et al, 2005 (88) | Word Color Interference Test (word and color trials) | Same as controls |
|  |  |  |  |
| **General cognitive functioning** |  |  |  |
|  | Cavedini et al, 2002 (50) | Weigl’s sorting test, WSCT | Same as controls |
|  | Brand et al, 2005 (88) | DemTect | Same as controls |
|  | Goudriaan et al, 2006 (34) | SST mean reaction time, WAIS Digit Span Forwards, Benton Visual Retention Test, Sorting task of the Groningen Intelligence Test | Same as controls |
|  | Kalechstein et al, 2007 (35) | National American Reading Test | Same estimated premorbid intellectual functioning |
|  | Forbush et al, 2008 (41) | WAIS, Wide Range Achievement Test-3 Reading Scale | **Lower verbal intelligence than controls**  Same full IQ, performance IQ and reading achievement as controls |
|  | Ledgerwood et al, 2009 (66) | Shipley Institute of Living Scale | Same as controls |
|  |  |  |  |
|  |  |  |  |
| **MEMORY / WORKING MEMORY** |  |  |  |
|  | Goudriaan et al, 2006 (34) | Self-Ordered Pointing Task-abstract designs, WAIS Digit Span Forward and Backward | Same as controls |
|  | Leiserson et al, 2007 (64) | Ordered Pointing Task, Spatial and Non-Spatial Conditional Association Tasks | **Lower performance than controls (on the Conditional Association Tasks : deficits in working memory)**  Same as controls (on the Ordered Pointing Task) |
|  | Forbush et al, 2008 (41) | Letter-Number Sequencing subtest (WAIS) | **Lower performance than controls** |
|  | Marazziti et al, 2008 (119) | Wechsler Memory Scale R | Same as controls |
|  | Ledgerwood et al, 2009 (66) | Immediate and Delayed Memory Tasks, Paced Auditory Serial Addition Task | Same as controls |
|  | Ledgerwood et al, 2012 (55) | Wechsler Memory Scale III | Same as controls |
|  | Albein-Urios et al, 2012 (33) | N-back | Same as controls |
|  | Hur et al, 2012 (42) | California Verbal Learning Test (Korean version), Rey-Osterrieth Complex Figure Test (recall condition) | Same as controls |
|  | Brevers et al, 2013 (47) | Operation-span Task | **Lower performance than controls** |
|  |  |  |  |
|  |  |  |  |
| **ATTENTION** |  |  |  |
|  | Vizcaino et al, 2013 (43) | Visual Probe Task with exposure times that assess maintenance of attention | **Attentional bias for gambling cues (not in controls)**  **Slower reaction times than controls, whatever the condition** |
|  | Lorains et al, 2014 (44) | Addiction Stroop task, Sustained Attention to Response Task (SART)  SST (number of errors to the Go trials) | Same as controls  **More errors to the Go trials than controls (inattention)** |
|  |  |  |  |

|  |  |  |  |
| --- | --- | --- | --- |
| **CUE-REACTIVITY FOR GAMBLING CUES** |  |  |  |
|  | Sharpe et al, 1995 (45) | Five cue-reactivity tasks combined with SCL, frontalis EMG and EKG measures:   - Neutral (reciting the alphabet) - Horse-race video - Poker machine video - Poker machine video with a cognitive distraction task - Imaginal poker machine winning situation | **Higher increase of arousal in response to gambling-related cues (especially video cues), compared to controls**  **Higher influence of cognitive distraction (limitation of the increase in arousal), compared to control** |
|  |  |  |  |
|  |  |  |  |
| **METACOGNITION** |  |  |  |
|  | Goodie, 2005 (48) | Betting-on-confidence task (under uncertainty and outside of a gambling context) | **Compared to controls, lower influence of perceived control on betting decisions but higher influence on correlation between confidence and bet acceptance, greater overall overconfidence and bet acceptance** |
|  | Brevers et al, 2013 (47) | IGT with post-decision wagering | Same post-decision wagering on advantageous decks as controls  **Higher post-decision wagering on disadvantageous decks than controls** |
|  |  |  |  |
|  |  |  |  |
| **EXECUTIVE FUNCTIONING** |  |  |  |
| **Response inhibition** |  |  |  |
| - Cognitive inhibition | Potenza et al, 2003 (59) | Classical Stroop task coupled with fMRI | Same as controls |
|  | Kertzman et al, 2006 (116) | Classical Stroop task (reverse variant) | **Lower performance than controls** |
|  | Goudriaan et al, 2006 (34) | Classical Stroop task | **Lower performance than controls** |
|  | Kalechstein et al, 2007 (35) | Classical Stroop task | **Lower performance than controls** |
|  | Forbush et al, 2008 (41) | Classical Stroop task | **Lower performance than controls** |
|  | Alvarez-Moya et al, 2009 (58) | Classical Stroop task | Same as controls (but trend for higher interference) |
|  | Molde et al, 2010 (81) | Classical Stroop task (pictorial variant) | **Lower performance than controls** |
|  | Kertzman et al, 2011 (53) | Classical Stroop task (reverse variant) | **Lower performance than controls** |
|  | Hur et al, 2012 (42) | Classical Stroop task | Same as controls |
|  | Albein-Urios et al, 2012 (33) | Classical Stroop task | **Lower performance than controls** |
|  | Ledgerwood et al, 2012 (55) | Classical Stroop task | Same as controls |
|  | Lorains et al, 2014 (44) | Addiction Stroop task, Sustained Attention to Response Task (SART), Random Number Generation Task (RNGT) | **Faster cycling through response set than controls (RNGT)**  Same as controls for the addiction Stroop task, SART and other parameters of the RNGT |
|  |  |  |  |
| - Motor inhibition | Goudriaan et al, 2005 (51) | Go/No-Go discrimination task (reward and loss version) | **Lower performance than controls**  **Faster response after loss than after reward compared to slower responses after loss than after reward for controls** |
|  | Fuentes et al, 2006 (62) | Simple Choice Auditory and Visual Reaction tasks (Go/No-Go tasks) | **More errors than controls**  Same response times as controls |
|  | Goudriaan et al, 2006 (34) | SST, Circle Tracing Task | **Lower performance than controls** |
|  | Leiserson et al, 2007 (64) | Go/No-Go (reward-punishment version) | Same as controls |
|  | Roca et al, 2008 (57) | Go/No-Go | **Lower performance than controls** |
|  | Kertzman et al, 2008 (63) | Go/No-Go, CPT | **Lower performance than controls** |
|  | Ledgerwood et al, 2009 (66) | GoStop Impulsivity Paradigm | Same as controls |
|  | Odlaug et al, 2011 (67) | SST | **Lower performance than controls** |
|  | Kertzman et al, 2011 (53) | Go/No-Go, CPT | **Lower performance than controls** |
|  | Ledgerwood et al, 2012 (55) | GoStop Impulsivity Paradigm | Same as controls |
|  | De Ruiter et al, 2012 (65) | SST (accuracy and reaction times) | Same as controls |
|  | Grant et al, 2012 (69) | SST | **Lower performance than controls** |
|  | Billieux et al, 2012 (68) | Go-stop impulsivity paradigm | **Lower performance than controls** |
|  | Lorains et al, 2014 (44) | SST | Same response times as controls |
|  |  |  |  |
| **Concept generation / abstraction** |  |  |  |
|  | Goudriaan et al, 2005 (51) | IGT (conceptual knowledge) | **Lower performance than controls** |
|  | Goudriaan et al, 2006 (34) | WCST (number of categories completed) | **Lower performance than controls** |
|  | Marazziti et al, 2008 (119) | WCST (number of categories completed, failure to maintain and learning-to-learn score) | Same as controls (number of categories completed)  **Lower performance than controls (failure to maintain and learning-to-learn score)** |
|  | Forbush et al, 2008 (41) | WCST (number of categories completed, non-perseverative errors and learning-to-learn score) | Same as controls (number of categories completed and non-perseverative errors)  **Lower performance than controls (learning-to-learn score)** |
|  | Alvarez-Moya et al, 2009 (58) | WCST (number of categories completed, number of trials to first category, number of trials administered, % of conceptual-level responses) | Same as controls (number of categories completed, trials to first category)  **Lower performance than controls (number of trials administered, % of conceptual-level responses)** |
|  | Hur et al, 2012 (42) | WSCT (number of categories completed and non-perseverative errors) | **Higher number of non-perseverative errors than controls**  Same number of categories completed as controls |
|  | Ledgerwood et al, 2012 (55) | WCST (number of categories completed) | Same as controls (when controlling for IQ) |
|  |  |  |  |
| **Planning** | Goudriaan et al, 2006 (34) | Tower of London | **Lower performance than controls** |
|  | Ledgerwood et al, 2012 (55) | Tower of London | **Lower performance than controls** |
|  |  |  |  |
| **Time estimation** | Goudriaan et al, 2006 (34) | Time estimation and reproduction tests | **Lower performance than controls** |
|  |  |  |  |
| **Flexibility** |  |  |  |
| - Set shifting / perseveration (reactive flexibility) | Goudriaan et al, 2006 (34) | WCST (perseverative errors) | Same as controls |
|  | Kalechstein et al, 2007 (35) | TMT part B | **Lower performance than controls** |
|  | Forbush et al, 2008 (41) | TMT, WSCT (perseverative errors) | Same as controls for TMT  **Lower performance than controls for WCST** |
|  | Marazziti et al, 2008 (119) | WCST (perseverative errors) | **Lower performance than controls** |
|  | Alvarez-Moya et al, 2009 (58) | WCST (perseverative errors) | **Lower performance than controls** |
|  | Odlaug et al, 2011 (67) | Intra-dimensional/extra-dimensional set shift task | **Lower performance than controls** |
|  | Grant et al, 2012 (69) | Intra-dimensional/extra-dimensional set shift task | **Lower performance than controls** |
|  | Hur et al, 2012 (42) | TMT, WSCT (perseverative errors) | Same as controls |
|  | Ledgerwood et al, 2012 (55) | WSCT (perseverative errors) | Same as controls |
|  | Albein-Urios et al, 2012 (33) | Stroop task (shifting condition) | Same as controls |
|  |  |  |  |
| - Fluency (spontaneous flexibility) | Goudriaan et al, 2006 (34) | COWAT | **Lower performance than controls** |
|  | Kalechstein et al, 2007 (35) | Ruff Figural Fluency Test | **Lower performance than controls** |
|  | Forbush et al, 2008 (41) | COWAT, Boston Diagnostic Aphasia Examination Animal Naming Test | **Lower performance than controls** |
|  | Marazziti et al, 2008 (119) | Verbal Associative Fluency Test | Same as controls |
|  | Hur et al, 2012 (42) | COWAT, Category Fluency Test | Same as controls |
|  | Ledgerwood et al, 2012 (55) | COWAT | Same as controls |
|  |  |  |  |
| **Decision-making** |  |  |  |
| - General | Kertzman et al, 2010 (120) | MFFT (computerized variant) with instructions for accuracy (no time pressure for responding) | **Lower performance than controls**  Same speed-accuracy tradeoff as controls |
| - Delay discounting | Petry, 2001 (84) | Delay Discounting Paradigm | **Lower ability to delay rewards than controls** |
|  | Dixon et al, 2003 (82) | Hypothetical choice task | **Lower ability to delay rewards than controls** |
|  | Ledgerwood et al, 2009 (66) | Delayed Discounting of Monetary Rewards task Single Key Impulsivity Paradigm | **Lower ability to delay rewards than controls**  Same as controls |
|  | Billieux et al, 2012 (68) | Single Key Impulsivity Paradigm | **Lower ability to delay rewards than controls** |
|  | Kraplin et al, 2014 (83) | Delay Discounting Paradigm | **Lower ability to delay rewards than controls** |
|  |  |  |  |
| - Use of feedback for future decisions | Brand et al, 2005 (88) | Game of Dice Task | **Lower use of negative feedback than controls** |
|  | Goudriaan et al, 2005 (51) | IGT - response speed and shifting after rewards/losses  Card Playing Task - response speed and shifting after rewards/losses | **No higher switch of deck selection after loss than after reward compared to controls**  **No slower response speed after loss than after reward compared to controls** |
|  | Hewig et al, 2010 (85) | Black Jack task (17 & 4) | **Higher risky decisions after prior losses** |
|  | Brevers et al, 2014 (49) | IGT - recency parameters | Same as controls |
|  | Lorains et al, 2014 (56) | IGT - recency parameters | Same as controls |
|  |  |  |  |
| - Sensitivity to monetary reward and punishment | Sharpe, 2004 (86) | Imaginal “win” or “lose” tasks combined with SCL measure | **No higher arousal for winning than for loosing (equal arousal for win and lose conditions) compared to controls**  **More subjective excitement for both winning and losing, and lower subjective tense for losing for PG** |
|  | Goudriaan et al, 2006 (52) | IGT combined with ECG (HR) and SCL measure | **Lower physiological reward sensitivity than controls** |
|  | Hewig et al, 2010 (85) | Black Jack task with EEG (calculation of event-related brain potential in response to wins and losses) | **Higher sensitivity to rewards rather than insensitivity to losses** |
|  | Kreussel et al, 2013 (87) | Black Jack task with EEG (calculation of event-related brain potential in response to near and full losses) | Near losses perceived more negatively than full losses in later stage of evaluation processing, as for controls  **Near losses not perceived more negatively than full losses contrary to controls, at early stage of evaluation processing**  Lower risk taking after near losses, as for controls |
|  | Brevers et al, 2014 (49) | IGT - gain/loss parameter | **Higher sensitivity to gains than controls** |
|  | Fauth-Buhler et al, 2014 (71) | Effort-dependent instrumental-motivation task without any gambling-like features | Same as controls (outside a gambling context) |
|  | Lorains et al, 2014 (56) | IGT - gain/loss and utility shape parameters, Loss Aversion Task | **Higher attention to gains than controls**  **Lower loss aversion than controls** |
|  |  |  |  |
| - Decision-making under risk and/or under ambiguity/uncertainty | Cavedini et al, 2002 (50) | IGT (under ambiguity / risk) | **Lower total performance and no shift toward advantageous card selection during the task compared to controls** |
|  | Brand et al, 2005 (88) | Game of Dice Task (under risk) | **Lower performance than controls** |
|  | Goodie, 2005 (48) | Betting-on-confidence task (under uncertainty and outside of a gambling context) | **Lower performance than controls** |
|  | Goudriaan et al, 2005 (51) | IGT (under ambiguity / risk)  Card Playing Task (under risk) | **Lower total performance and no shift toward advantageous card selection during the task compared to controls**  **Lower performance than controls (perseverative deficits)** |
|  | Goudriaan et al, 2006 (52) | IGT (under ambiguity / risk) | **No shift toward advantageous card selection during the task compared to controls** |
|  | Linnet et al, 2006 (70) | Mouse game (modified version of the IGT without monetary rewards) | **Lower performance than controls** |
|  | Labudda et al, 2007 (118) | Game of Dice Task (under risk) | **Lower performance than controls** |
|  | Lakey et al, 2007 (54) | Georgia Gamblign Task (under risk, overconfidence), IGT (under ambiguity / risk) | **Lower performance than controls** |
|  | Forbush et al, 2008 (41) | IGT (under ambiguity / risk) | **No shift toward advantageous card selection during the task compared to controls** |
|  | Roca et al, 2008 (57) | IGT (under ambiguity / risk) | **Lower total performance and no shift toward advantageous card selection during the task compared to controls** |
|  | Ledgerwood et al, 2009 (66) | Baloon Analogue Risk Task (under risk and outside of a gambling context) | Same as controls (outside a gambling context) |
|  | Kertzman et al, 2011 (53) | IGT (under ambiguity / risk) | **Lower total performance and no shift toward advantageous card selection during the task compared to controls** |
|  | Ledgerwood et al, 2012 (55) | IGT (under ambiguity / risk) | **Lower performance than controls** |
|  | Brevers et al, 2013 (47) | IGT with post-decision wagering | **Lower total performance and no shift toward advantageous card selection during the task compared to controls** |
|  | Brevers et al, 2014 (49) | IGT (under ambiguity / risk) total performance + consistency parameters | **Lower total performance and no shift toward advantageous card selection during the task compared to controls**  Same consistency as controls |
|  | Kraplin et al, 2014 (83) | CGT (under risk) | **Higher risk taking behavior than controls** |
|  | Lorains et al, 2014 (56) | IGT (under ambiguity / risk) total performance + consistency parameters | **Lower performance than controls**  Same shift toward advantageous card selection during the task as controls  **Less consistency (more random or erratic choices) than controls** |
|  |  |  |  |
| - Anticipatory somatic markers of decision-making (risk assessment) | Goudriaan et al, 2006 (52) | IGT combined with ECG and SCL measure | **Impaired risk assessment** |
|  | Labudda et al, 2007 (118) | Game of Dice Task combined with salivary cortisol and alpha-amylase levels | **Correlation of alpha-amylase levels (decrease) with disadvantageous choices but no for controls** |
|  |  |  |  |
|  |  |  |  |
| **SOCIAL COGNITION** | Kornreich et al, 2016 (72) | Three emotion recognition tasks (musical, vocal and facial) | **Underestimation of peacefulness in music compared to controls**  **Lower accuracy for reading voices and faces than controls**  **Overestimation of emotional intensity in neutral voices and faces** |
| **VISUO-SPATIAL & VISUO-CONSTRUCTIVE ABILITIES** | Forbush et al, 2008 (41) | Picture Completion subtest (WAIS) | **Lower performance than controls** |
|  | Hur et al, 2012 (42) | Rey-Osterrieth Complex Figure Test (copy condition) | **More fragmentation and less organization** |
|  |  |  |  |

* as the review focuses only on the potential neurocognitive targets of CR interventions for GD management, the aim was to identify neurocognitive alterations/biases for PG/ PrG compared to controls, so that only studies comparing PG or PrG to HC are reported in this synthesis table.

Within each category, studies are reported in chronological order.

Bold font indicates an alteration/bias for PG/PrG compared to controls in the cognitive function assessed

Normal font indicates no alteration/bias for PG/PrG compared to controls in the cognitive function assessed

**PG**: pathological gamblers/gambling (diagnosed with DSM or ICD criteria); **PrG**: problem/excessive gamblers/gambling (identified with a SOGS scores ≥ 5 or a CPGI ≥ 8); **HC**: Healthy controls; **OCD**: Obsessive-Compulsive Disorder; **ADHD**: Attention Deficit Hyperactivity Disorder; **PTSD**: post-traumatic stress disorder

**UPPS**: UPPS impulsive behavior scale; **TCI-R**: Temperament and Character Inventory Revised; **SOGS**: South Oaks Gambling Screen, **VAS**: Visual Analog Scale; **CPGI**: Canadian Problem Gambling Index

**WSCT**: Wisconsin Card Sorting Test (Modified version: M-WCST); **TMT**: Trail Making Test; **IGT**: Iowa Gambling Task (ABCD-EFGH version for the assessment of sensitivity to reward and punishment respectively); **CGT**: Cambridge Gambling Task; **COWAT**: Controlled Oral Word Association Test; **CPT**: Continuous Performance Test; **SST**: Stop-Signal Task; **WAIS**: Wechsler Abbreviated Intelligence Scale; **MFFT**: Matching Familiar Figures Test; **IDED set-shift test**: Intra-dimensional/extra-dimensional set-shift test

**EKG**: Electrocardiogram; **SCL**: Skin Conductance Level; **EEG**: Electroencephalogram; **EOG**: Electro-oculogram; **EMG**: Electromyogram

**Table 3 - Main CR programs used for the treatment of alcohol-use disorders and comparison with Playmancer**

| **Names of the CR programs** | **CR techniques used** | **Disorder(s)** | **Number of sessions** | **Duration of sessions** | **Follow-up** | **Format** | **Individual vs group sessions** | **Setting: Inpatient vs outpatient** |
| --- | --- | --- | --- | --- | --- | --- | --- | --- |
| Serious Video Game PlayMancer for emotional regulation training (14) | Serious video-game with biofeedback (reduction of arousal and improvement of decision-making and planning) | - Bulimia Nervosa (BN)  - Gambling Disorder (GD) | - 9 (BN)  - 10 (GD) | Exposure to the video-game while the performance of the patient is collected during 20 min. Relaxing music played for 3 min before and after the video-game. | No | Computerized (adventure-stimulation game) | Individual | Outpatient |
| Multiple methods in cognitive retraining (123) | Multiple (attention, information processing, memory) | Alcohol use disorder (without associated neuropsychiatric disorder) | Daily during 6 weeks | 1 hour per session | No | Computerized and "paper and pencil" | Individual | Outpatient |
| Goldstein rehabilitation training procedures (124) | Multiple (selective attention, speed of information processing during decision-making, psychomotor and information processing speed, spatial-constructional ability and its underlying analytical reasoning basis) | Alcohol use disorder (with associated neuropsychiatric disorder) | 5 sessions over a 3-week period | 30 minutes per session | No | Paper and pencil | Individual | Inpatient |
| Cogpack software (german-speaking countries) (27) | Multiple (attention, executive function, memory) | Alcohol use disorder | 12 sessions (3 per week) | 45 - 60 minutes | No | Computerized | Individual | Inpatient |
| Working memory training adapted from Klingberg (125) | Working memory training (visuospatial WM task, backward digit span task, letter span task, with difficulty level automatically adjusted) | Alcohol use disorder (problem drinkers) | 25 sessions over 25 days | The length of the sequences increases and decreases accord- ing to participants’ performance | 1 month follow-up | Computerized | Individual | Outpatient |
| AACTP (Alcohol Attention-Control Training Program)  (126) | Restorative training focused on attentional biases and inhibitory processes (based on a pictorial alcohol Stroop task) | Alcohol use disorder (hazardous and harmful drinkers) | 2 sessions for hazardous drinkers and 4 sessions for harmful drinkers | 2 full sets for each session with time limits (?); each set includes 3 tasks interspersed by 5 minutes.  Rest for 10 minutes between the 2 full sets. | 3-months follow-up | Computerized | Individual | Outpatient |
| Attentional Bias Modification training (100) | Restorative training focused on attentional biases and inhibitory processes (Modified visual-probe task) | Alcohol use disorder (has been also used in anxiety) | 5 sessions | Approximately 30 minutes | 3-months follow-up | Computerized | Individual | Outpatient |
| Alcohol approach / avoidance task (alcohol-AAT) (106) | Approach bias (Cognitive bias) : Alcohol approach / avoidance task (alcohol-AAT) | Alcohol use disorder | 4 sessions on 4 consecutive days | 15 min per session | 1-year follow-up | Computerized | Individual | Inpatient |

**Bibliographic references**

14. Tarrega S, Castro-Carreras L, Fernandez-Aranda F, Granero R, Giner-Bartolome C, Aymami N, et al., A Serious Videogame as an Additional Therapy Tool for Training Emotional Regulation and Impulsivity Control in Severe Gambling Disorder*.* *Frontiers in Psychology* (2015) **6**. doi: 10.3389/fpsyg.2015.01721

27. Rupp CI, Kemmler G, Kurz M, Hinterhuber H, and Fleischhacker WW, Cognitive remediation therapy during treatment for alcohol dependence*.* *Journal of studies on alcohol and drugs* (2012) **73**: 625-34. doi: 10.15288/jsad.2012.73.625

33. Albein-Urios N, Martinez-Gonzalez JM, Lozano O, Clark L, and Verdejo-Garcia A, Comparison of impulsivity and working memory in cocaine addiction and pathological gambling: Implications for cocaine-induced neurotoxicity*.* *Drug and Alcohol Dependence* (2012) **126**: 1-6. doi: 10.1016/j.drugalcdep.2012.03.008

34. Goudriaan AE, Oosterlaan J, de Beurs E, and van den Brink W, Neurocognitive functions in pathological gambling: a comparison with alcohol dependence, Tourette syndrome and normal controls*.* *Addiction* (2006) **101**: 534-47. doi: 10.1111/j.1360-0443.2006.01380.x

35. Kalechstein AD, Fong T, Rosenthal RJ, Davis A, Vanyo H, and Newton TF, Pathological gamblers demonstrate frontal lobe impairment consistent with that of methamphetamine-dependent individuals*.* *The Journal of neuropsychiatry and clinical neurosciences* (2007) **19**: 298-303. doi: 10.1176/jnp.2007.19.3.298

40. Goudriaan AE, Oosterlaan J, De Beurs E, and Van Den Brink W, The role of self-reported impulsivity and reward sensitivity versus neurocognitive measures of disinhibition and decision-making in the prediction of relapse in pathological gamblers*.* *Psychological Medecine* (2008) **38**: 41-50. doi: 10.1017/S0033291707000694

41. Forbush KT, Shaw M, Graeber MA, Hovick L, Meyer VJ, Moser DJ, et al., Neuropsychological characteristics and personality traits in pathological gambling*.* *CNS Spectrum* (2008) **13**: 306-15.

42. Hur JW, Shin N, Kim SN, Jang JH, Choi JS, Shin YC, et al., Do pathological gambling and obsessive-compulsive disorder overlap? a neurocognitive perspective*.* *CNS Spectrum* (2012) **17**: 207-13. doi: 10.1017/S1092852912000545.

43. Vizcaino EJ, Fernandez-Navarro P, Blanco C, Ponce G, Navio M, Moratti S, et al., Maintenance of attention and pathological gambling*.* *Psychology of addictive behaviors* (2013) **27**: 861-7. doi: 10.1037/a0032656

44. Lorains FK, Stout JC, Bradshaw JL, Dowling NA, and Enticott PG, Self-reported impulsivity and inhibitory control in problem gamblers*.* *Journal of clinical and experimental neuropsychology* (2014) **36**: 144-57. doi: 10.1080/13803395.2013.873773

45. Sharpe L, Tarrier N, Schotte D, and Spence SH, The role of autonomic arousal in problem gambling*.* *Addiction* (1995) **90**: 1529-40. doi: 10.1046/j.1360-0443.1995.9011152911.x

47. Brevers D, Cleeremans A, Bechara A, Greisen M, Kornreich C, Verbanck P, et al., Impaired self-awareness in pathological gamblers*.* *Journal of Gambling Studies* (2013) **29**: 119-29. doi: 10.1007/s10899-012-9292-2.

48. Goodie AS, The role of perceived control and overconfidence in pathological gambling*.* *Journal of gambling studies* (2005) **21**: 481-502. doi: 10.1007/s10899-005-5559-1

49. Brevers D, Koritzky G, Bechara A, and Noel X, Cognitive processes underlying impaired decision-making under uncertainty in gambling disorder*.* *Addictive Behavaviors* (2014) **39**: 1533-6. doi: doi.org/10.1016/j.addbeh.2014.06.004

50. Cavedini P, Riboldi G, Keller R, D'Annucci A, and Bellodi L, Frontal lobe dysfunction in pathological gambling patients*.* *Biological psychiatry* (2002) **51**: 334-41. doi: 10.1016/S0006-3223(01)01227-6

51. Goudriaan AE, Oosterlaan J, de Beurs E, and van den Brink W, Decision making in pathological gambling: a comparison between pathological gamblers, alcohol dependents, persons with Tourette syndrome, and normal controls*.* *Brain Research Cognitive Brain Research* (2005) **23**: 137-51. doi: 10.1016/jcogbrainres.2005.01.017

52. Goudriaan AE, Oosterlaan J, de Beurs E, and van den Brink W, Psychophysiological determinants and concomitants of deficient decision making in pathological gamblers*.* *Drug and Alcohol Dependence* (2006) **84**: 231-9. doi: 10.1016/j.drugalcdep.2006.02.007

53. Kertzman S, Lidogoster H, Aizer A, Kotler M, and Dannon PN, Risk-taking decisions in pathological gamblers is not a result of their impaired inhibition ability*.* *Psychiatry Research* (2011) **188**: 71-7. doi: 10.1016/j.psychres.2011.02.021

54. Lakey CE, Goodie AS, and Campbell WK, Frequent card playing and pathological gambling: the utility of the Georgia Gambling Task and Iowa Gambling Task for predicting pathology*.* *Journal of gambling studies* (2007) **23**: 285-97. doi: 10.1007/s10899-006-9034-4

55. Ledgerwood DM, Orr ES, Kaploun KA, Milosevic A, Frisch GR, Rupcich N, et al., Executive function in pathological gamblers and healthy controls*.* *Journal of gambling studies* (2012) **28**: 89-103. doi: 10.1007/s10899-010-9237-6

56. Lorains FK, Dowling NA, Enticott PG, Bradshaw JL, Trueblood JS, and Stout JC, Strategic and non-strategic problem gamblers differ on decision-making under risk and ambiguity*.* *Addiction* (2014) **109**: 1128-37. doi: 10.1111/add.12494

57. Roca M, Torralva T, Lopez P, Cetkovich M, Clark L, and Manes F, Executive functions in pathologic gamblers selected in an ecologic setting*.* *Cognitive and Behavioral Neurology* (2008) **21**: 1-4. doi: 10.1097/WNN.0b013e3181684358

58. Alvarez-Moya EM, Jimenez-Murcia S, Moragas L, Gomez-Pena M, Aymami MN, Ochoa C, et al., Executive functioning among female pathological gambling and bulimia nervosa patients: preliminary findings*.* *Journal of the International Neuropsychological Society : JINS* (2009) **15**: 302-6. doi: 10.1017/S1355617709090377.

59. Potenza MN, Leung H-C, Blumberg HP, Peterson BS, Fulbright RK, Lacadie CM, et al., An fMRI Stroop Task Study of Ventromedial Prefrontal Cortical Function in Pathological Gamblers*.* *The American Journal of Psychiatry* (2003) **160**: 1990-1994. doi: 10.1176/appi.ajp.160.11.1990

62. Fuentes D, Tavares H, Artes R, and Gorenstein C, Self-reported and neuropsychological measures of impulsivity in pathological gambling*.* *Journal of the International Neuropsychological Society* (2006) **12**: 907-12. doi: 10.1017/S1355617706061061

63. Kertzman S, Lowengrub K, Aizer A, Vainder M, Kotler M, and Dannon PN, Go-no-go performance in pathological gamblers*.* *Psychiatry Research* (2008) **161**: 1-10. doi: 10.1016/j.psychres.2007.06.026

64. Leiserson V and Pihl RO, Reward-sensitivity, inhibition of reward-seeking, and dorsolateral prefrontal working memory function in problem gamblers not in treatment*.* *Journal of gambling studies* (2007) **23**: 435-55. doi: 10.1007/s10899-007-9065-5

65. de Ruiter MB, Oosterlaan J, Veltman DJ, van den Brink W, and Goudriaan AE, Similar hyporesponsiveness of the dorsomedial prefrontal cortex in problem gamblers and heavy smokers during an inhibitory control task*.* *Drug and alcohol dependence* (2012) **121**: 81-9. doi: 10.1016/j.drugalcdep.2011.08.010.

66. Ledgerwood DM, Alessi SM, Phoenix N, and Petry NM, Behavioral assessment of impulsivity in pathological gamblers with and without substance use disorder histories versus healthy controls*.* *Drug and alcohol dependence* (2009) **105**: 89-96. doi: 10.1016/j.drugalcdep.2009.06.011

67. Odlaug BL, Chamberlain SR, Kim SW, Schreiber LR, and Grant JE, A neurocognitive comparison of cognitive flexibility and response inhibition in gamblers with varying degrees of clinical severity*.* *Psychological medicine* (2011) **41**: 2111-9. doi: 10.1017/S0033291711000316

68. Billieux J, Lagrange G, Van der Linden M, Lancon C, Adida M, and Jeanningros R, Investigation of impulsivity in a sample of treatment-seeking pathological gamblers: a multidimensional perspective*.* *Psychiatry Research* (2012) **198**: 291-6. doi: 10.1016/j.psychres.2012.01.001

69. Grant JE, Odlaug BL, Chamberlain SR, and Schreiber LR, Neurocognitive dysfunction in strategic and non-strategic gamblers*.* *Progress in neuro-psychopharmacology and biological psychiatry* (2012) **38**: 336-40. doi: 10.1016/j.pnpbp.2012.05.006

70. Linnet J, Rojskjaer S, Nygaard J, and Maher BA, Episodic chasing in pathological gamblers using the Iowa gambling task*.* *Scandinavian journal of psychology* (2006) **47**: 43-9. doi: 10.1111/j.1467-9450.2006.00491.x

71. Fauth-Buhler M, Zois E, Vollstadt-Klein S, Lemenager T, Beutel M, and Mann K, Insula and striatum activity in effort-related monetary reward processing in gambling disorder: the role of depressive symptomatology*.* *NeuroImage. Clinical* (2014) **6**: 243-51. doi: 10.1016/j.nicl.2014.09.008

72. Kornreich C, Saeremans M, Delwarte J, Noel X, Campanella S, Verbanck P, et al., Impaired non-verbal emotion processing in Pathological Gamblers*.* *Psychiatry Research* (2016) **236**: 125-9. doi: 10.1016/j.psychres.2015.12.020

81. Molde H, Pallesen S, Sætrevik B, Hammerborg D, Laberg J, and Johnsen B, Attentional biases among pathological gamblers*.* *International Gambling Studies* (2010) **10**: 45-59. doi: 10.1080/14459791003652501

82. Dixon MR, Marley J, and Jacobs EA, Delay discounting by pathological gamblers*.* *Journal of Applied Behavior Analysis* (2003) **36**: 449-458. doi: 10.1901/jaba.2003.36-449

83. Kraplin A, Dshemuchadse M, Behrendt S, Scherbaum S, Goschke T, and Buhringer G, Dysfunctional decision-making in pathological gambling: pattern specificity and the role of impulsivity*.* *Psychiatry Research* (2014) **215**: 675-82. doi: 10.1016/j.psychres.2013.12.041.

84. Petry NM, Pathological gamblers, with and without substance use disorders, discount delayed rewards at high rates*.* *Journal of Abnormal Psychology* (2001) **110**: 482-7.

85. Hewig J, Kretschmer N, Trippe RH, Hecht H, Coles MG, Holroyd CB, et al., Hypersensitivity to reward in problem gamblers*.* *Biological psychiatry* (2010) **67**: 781-3. doi: 10.1016/j.biopsych.2009.11.009

86. Sharpe L, Patterns of autonomic arousal in imaginal situations of winning and losing in problem gambling*.* *Journal of gambling studies* (2004) **20**: 95-104. doi: 10.1023/B:JOGS.0000016706.96540.43

87. Kreussel L, Hewig J, Kretschmer N, Hecht H, Coles MG, and Miltner WH, How bad was it? Differences in the time course of sensitivity to the magnitude of loss in problem gamblers and controls*.* *Behavioural brain research* (2013) **247**: 140-5. doi: 10.1016/j.bbr.2013.03.024

88. Brand M, Kalbe E, Labudda K, Fujiwara E, Kessler J, and Markowitsch HJ, Decision-making impairments in patients with pathological gambling*.* *Psychiatry Research* (2005) **133**: 91-9. doi: 10.1016/j.psychres.2004.10.003

92. Alvarez-Moya EM, Ochoa C, Jimenez-Murcia S, Aymami MN, Gomez-Pena M, Fernandez-Aranda F, et al., Effect of executive functioning, decision-making and self-reported impulsivity on the treatment outcome of pathologic gambling*.* *Journal of Psychiatry & Neuroscience* (2011) **36**: 165-75. doi: 10.1503/jpn.090095

100. Schoenmakers TM, de Bruin M, Lux IF, Goertz AG, Van Kerkhof DH, and Wiers RW, Clinical effectiveness of attentional bias modification training in abstinent alcoholic patients*.* *Drug and alcohol dependence* (2010) **109**: 30-6. doi: 10.1016/j.drugalcdep.2009.11.022

106. Wiers RW, Eberl C, Rinck M, Becker ES, and Lindenmeyer J, Retraining automatic action tendencies changes alcoholic patients' approach bias for alcohol and improves treatment outcome*.* *Psychological science* (2011) **22**: 490-7. doi: 10.1177/0956797611400615

115. Alessi SM and Petry NM, Pathological gambling severity is associated with impulsivity in a delay discounting procedure*.* *Behavioural Processes* (2003) **64**: 345-354. doi: 10.1016/S0376-6357503°00150-5

116. Kertzman S, Lowengrub K, Aizer A, Nahum ZB, Kotler M, and Dannon PN, Stroop performance in pathological gamblers*.* *Psychiatry Research* (2006) **142**: 1-10. doi: 10.1016/j.psychres.2005.07.027

117. Rodriguez-Jimenez R, Avila C, Jimenez-Arriero MA, Ponce G, Monasor R, Jimenez M, et al., Impulsivity and sustained attention in pathological gamblers: influence of childhood ADHD history*.* *Journal of gambling studies* (2006) **22**: 451-61. doi: 10.1007/s10899-006-9028-2

118. Labudda K, Wolf OT, Markowitsch HJ, and Brand M, Decision-making and neuroendocrine responses in pathological gamblers*.* *Psychiatry Research* (2007) **153**: 233-43. doi: 10.1016/j.psychres.2007.02.002

119. Marazziti D, Catena Dell'osso M, Conversano C, Consoli G, Vivarelli L, Mungai F, et al., Executive function abnormalities in pathological gamblers*.* *Clinical Practice & Epidemiology in Mental Health* (2008) **4**: 7. doi: 10.1186/1745-0179-4-7

120. Kertzman S, Vainder M, Vishne T, Aizer A, Kotler M, and Dannon PN, Speed-accuracy tradeoff in decision-making performance among pathological gamblers*.* *European addiction research* (2010) **16**: 23-30. doi: 10.1159/000253861.

121. Grant JE, Chamberlain SR, Schreiber LR, and Odlaug BL, Gender-related clinical and neurocognitive differences in individuals seeking treatment for pathological gambling*.* *Journal of psychiatric research* (2012) **46**: 1206-11. doi: 10.1016/j.jpsychires.2012.05.013

122. Ochoa C, Alvarez-Moya EM, Penelo E, Aymami MN, Gomez-Pena M, Fernandez-Aranda F, et al., Decision-making deficits in pathological gambling: the role of executive functions, explicit knowledge and impulsivity in relation to decisions made under ambiguity and risk*.* *The American journal on addictions* (2013) **22**: 492-9. doi: 10.1111/j.1521-0391.2013.12061.x

123. Mathai G, Rao SL, and Gopinath PS, Neuropsychological rehabilitation of alcoholics: a preliminary report*.* *Indian Journal of Psychiatry* (1998) **40**: 280-288.

124. Goldstein G, Haas GL, Shemansky WJ, Barnett B, and Salmon-Cox S, Rehabilitation during alcohol detoxication in comorbid neuropsychiatric patients*.* *Journal of Rehabilitation Research and Development* (2005) **42**: 225-34.

125. Houben K, Wiers RW, and Jansen A, Getting a grip on drinking behavior: training working memory to reduce alcohol abuse*.* *Psychological Science* (2011) **22**: 968-75. doi: 10.1177/0956797611412392

126. Fadardi JS and Cox WM, Reversing the sequence: reducing alcohol consumption by overcoming alcohol attentional bias*.* *Drug and Alcohol Dependence* (2009) **101**: 137-45. doi: 10.1016/j.drugalcdep.2008.11.015
